# Active Magnetic-Field Stabilization with Atomic Magnetometer

**DOI:** 10.3390/s20154241

**Published:** 2020-07-30

**Authors:** Rui Zhang, Yudong Ding, Yucheng Yang, Zhaoyu Zheng, Jingbiao Chen, Xiang Peng, Teng Wu, Hong Guo

**Affiliations:** 1 College of Liberal Arts and Sciences, and Interdisciplinary Center for Quantum Information, National University of Defense Technology, Changsha 410073, China; zhangrui@nudt.edu.cn; 2 State Key Laboratory of Advanced Optical Communication Systems and Networks, Department of Electronics, and Center for Quantum Information Technology, Peking University, Beijing 100871, China; dyd@pku.edu.cn (Y.D.); yangyucheng@pku.edu.cn (Y.Y.); 1801213605@pku.edu.cn (Z.Z.); jbchen@pku.edu.cn (J.C.); xiangpeng@pku.edu.cn (X.P.); wuteng@pku.edu.cn (T.W.)

**Keywords:** magnetic field stabilization, unshielded, optically pumped magnetometers

## Abstract

A magnetically-quiet environment is important for detecting faint magnetic-field signals or nonmagnetic spin-dependent interactions. Passive magnetic shielding using layers of large magnetic-permeability materials is widely used to reduce the magnetic-field noise. The magnetic-field noise can also be actively monitored with magnetometers and then compensated, acting as a complementary method to the passive shielding. We present here a general model to quantitatively depict and optimize the performance of active magnetic-field stabilization and experimentally verify our model using optically-pumped atomic magnetometers. We experimentally demonstrate a magnetic-field noise rejection ratio of larger than ∼800 at low frequencies and an environment with a magnetic-field noise floor of ∼40 ${\mathrm{fT}/\mathrm{Hz}}^{1/2}$ in unshielded Earth’s field. The proposed model provides a general guidance on analyzing and improving the performance of active magnetic-field stabilization with magnetometers. This work offers the possibility of sensitive detections of magnetic-field signals in a variety of unshielded natural environments.

## 1. Introduction

A magnetically-quiet environment is of great demand for research areas that rely on detections of faint magnetic-field signals, such as magnetoencephalography [1,2,3,4], magnetocardiography [5,6,7], and ultralow-field nuclear magnetic resonance [8,9,10], where the amplitudes of the magnetic-field signals to be detected are typically several orders of magnitude smaller than the magnetic-field noise of the ambient environment. A magnetically-quiet environment is also important for the fundamental-physics experiments, such as measurements of permanent electric dipole moments (EDMs) [11,12,13], searches for exotic spin-dependent interactions mediated by hypothetical bosonic fields [14,15], and tests of fundamental symmetries [16,17,18], acting as an efficient way for reducing the systematic errors.

Passive magnetic shielding is widely used for suppressing the magnetic-field noise and is based on multi-layers of high magnetic permeability materials, such as mu-metal [19,20,21,22,23,24] and ferrite [25], or superconductors [26]. A magnetic shield using seven layers of mu-metal has a reported noise rejection ratio, a parameter used to describe the ability of the system in reducing the magnetic-field noise, of $10^{9}$ in the high-frequency region (around 1 kHz). The noise rejection ratio of the magnetic shield is smaller for lower frequencies and is 7.5$\times 10^{4}$ at 0.01 Hz [21]. Given that the environmental magnetic-field noise has an inverse-power-law frequency dependence and is higher at lower frequencies, the reduced performance of the passive magnetic shielding in suppressing the low-frequency magnetic-field noise needs to be compensated [27]. An efficient method for enhancing the noise rejection ratio in the low-frequency region is through gradiometric detection, i.e., using several identical magnetometers inside the magnetic shield and subtracting the measurement results of these magnetometers [28]

The noise rejection ratio in the low-frequency region can be improved by actively stabilizing the magnetic field [29,30,31]. The basic idea is to measure the magnetic-field noise with a magnetometer, compare the measured result with a reference, and generate an error signal to feedback control the current in a magnetic-field generator (solenoid or Helmholtz coil) and compensate for the magnetic-field noise. Many types of magnetometers have been used for active magnetic-field stabilization, including, but not limited to, optically-pumped atomic magnetometer (OPM) [32], superconducting quantum interference device (SQUID) [29], Hall sensor [33], fluxgate [34], and anisotropic magnetoresistive sensor [35]. In [21], the magnetic field outside the magnetic shield is monitored with SQUID sensors and actively stabilized, realizing a 30-fold improved noise rejection ratio of 2$\times 10^{6}$ at 0.01 Hz compared with that of the same magnetic shield without field stabilization.

Compared with passive magnetic shielding, the most attractive aspect of the active magnetic-field stabilization is the relatively low cost and large dimension. The demonstrated noise rejection ratio of the active magnetic-field stabilization is typically smaller than that of the magnetic shield and ranges from 10 to 1000 [6,29,33,36], depending on the noise level and response properties of the magnetometer and the parameter settings of the feedback controller. It is important to understand how these factors affect and how to improve the performance of active magnetic-field stabilization in a quantitative way. Considering that the parameter adjustment of magnetic-field stabilization is more or less based on the experience of researchers, in this work, we present a general model to quantitatively depict the performance of the active magnetic-field stabilization, from which we could derive the noise rejection ratio from the experimental coefficients, and experimentally verify the validity of our model using OPMs as the magnetic-field sensors. The proposed model is adopted to improve the noise rejection ratio of our active magnetic-field stabilization system and to produce a magnetically-quiet environment in unshielded Earth’s field. This approach can be extended to magnetic-field stabilization using different kinds of magnetometers, and can help researchers analyze their experimental systems quantitatively and optimize their systems in a targeted manner.

## 2. Experiment

**Figure 1:**
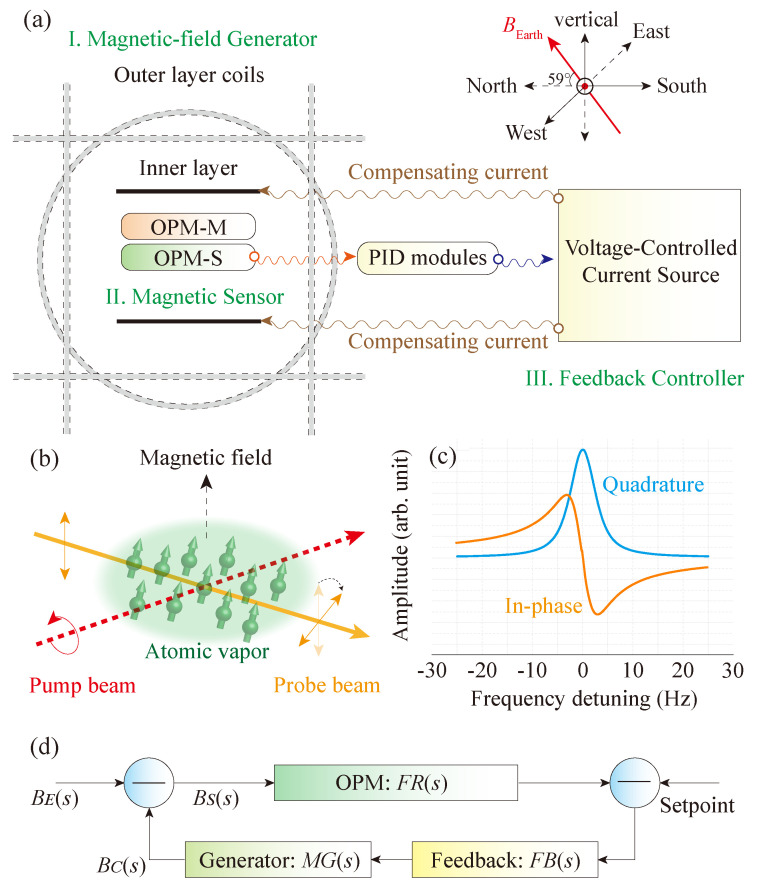
Active magnetic-field stabilization using optically-pumped atomic magnetometers (OPMs). (**a**) Schematics of the active magnetic-field stabilization system. The OPMs are placed in the center of two sets of Helmholtz coils. The readout from the bottom OPM sensor, OPM-S, is used as an input signal for the feedback controller (the PID modules and the voltage-controlled current source), which drives the inner layer of coils to compensate for the magnetic-field fluctuations. (**b**) Schematics of the OPM sensors. The Cs vapor cell is kept at room temperature. A circularly-polarized pump beam is amplitude modulated to synchronize the Larmor precession of the atomic spins. The optical rotation of a linearly-polarized probe beam is used to monitor the Larmor precession of the atomic spins. (**c**) A typical magnetic resonance signal of our OPM sensor in a 20,000 nT bias field. (**d**) The block diagram of the magnetic-field stabilization system. $B_{E}\left(s\right)$: the environmental magnetic-field variation in the Laplace domain, $B_{C}\left(s\right)$: the magnetic field produced by the inner layer of coils to actively compensate for the magnetic-field variation, $B_{S}\left(s\right)$: the noise-suppressed magnetic field and is equal to $B_{E}\left(s\right)-B_{C}\left(s\right)$, FR(s): the transfer function of the bottom OPM sensor, Setpoint: the target magnetic field to be stabilized to and is unchanged during the experiment, FB(s): the transfer function of the feedback controller, MG(s): the transfer function of the magnetic-field generator, or the inner layer of coils.

The apparatus of our active magnetic-field stabilization system is schematically shown in Figure 1a and consists of three basic components, i.e., a magnetic-field generator, a magnetic-field sensor, and a feedback controller. The magnetic-field generator in our system is two sets of coils. The outer layer of coils is for adjusting the strength of the magnetic field in the system, while the inner layer is for compensating the magnetic-field fluctuations. The outer layer is a set of three orthogonal coils, each of which has a diameter of 3 m. The current to magnetic-field conversion factors of the outer layer of coils are 60 nT/mA along the vertical direction and 32 nT/mA along the two horizontal directions, i.e., the north–south direction and the east–west direction. In this work, the direction of the magnetic field inside the coils is set to be along the vertical direction. To zero the horizontal components of the magnetic field, we measure the strength of the magnetic field along one horizontal direction with a fluxgate and adjust the current applied to the corresponding coils until the readout from the fluxgate becomes zero. We perform the same procedures for zeroing the magnetic field along the other horizontal direction. Repeating the above procedures several times reduces the horizontal components of the magnetic field inside the coils to be within ±10 nT, which is determined by the noise level of the fluxgate. We use a fluxgate in this step because we need a vector magnetometer to measure the horizontal components of the magnetic field, while our OPMs are scalar magnetometers that are only sensitive to the strength of the magnetic field. In the following steps, we use OPMs, which have higher sensitivities than that of the fluxgate, to stabilize the magnetic field and evaluate the magnetic-field noise after magnetic-field stabilization. The vertical component of the magnetic field can be set to any value below 10${}^{-4}$ T by adjusting the current in the outer layer of coils along the vertical direction. The direction of the magnetic field inside the coils is not limited to the vertical direction and can be rotated through adjusting the currents in the outer layer of coils. The inner layer is a pair of Helmholtz coils along the vertical direction and has a diameter of 0.3 m and a current to magnetic-field conversion factor of 35 nT/mA.

Figure 1b shows the basic structure of the OPM sensor used for magnetic-field stabilization (OPM-S) [37]. A circularly-polarized pump laser is used to polarize the cesium atoms in a self-made paraffin coated vapor cell [38]. The central frequency of the pump laser is locked to the D1 transition line of cesium (from 6${}^{2}S_{1/2}\;F=3$ to $6^{2}P_{1/2}\;F^{\prime }=4$, where *F* and $F^{\prime }$ are magnitudes of the total angular momentum). The intensity of the pump laser is modulated at an optimized duty cycle of 20% with an acoustic-optical modulator (AOM). The averaged power of the pump laser beam ($1/e^{2}$ diameter: ∼2.7 mm) is ∼50 μW. The modulation frequency is tuned around the Larmor frequency, which is proportional to the strength of the bias magnetic field. A macroscopic atomic magnetic moment is thus generated, of which the amplitude depends on the difference between the modulation frequency and the Larmor frequency and is maximum when the frequency difference is zero [39,40,41]. The generated macroscopic atomic magnetic moment precesses at the Larmor frequency under the influence of the bias magnetic field and modifies the absorptive and dispersive properties of the atomic vapor periodically. We use a linearly-polarized laser beam to transmit through the atomic vapor and extract the precession frequency of the macroscopic atomic magnetic moment through monitoring the polarization rotation [42]. To reduce the optical-pumping effect from the probe laser beam, the central frequency of the probe laser is positively detuned by ∼400 MHz from the D2 transition line (from 6${}^{2}S_{1/2}\;F=4$ to $6^{2}P_{1/2}\;F^{\prime }=5$) and the optimized power ($1/e^{2}$ diameter: ∼1.1 mm) is 50 μW. To suppress technical noises induced by the fluctuations of laser frequency and power, we actively stabilize the frequencies of both the pump and probe beams with Dichroic Atomic Vapor Laser Lock (DAVLL), and actively stabilize the averaged power of both the pump and probe beams that enter the cesium atomic vapor cell.

The cesium atomic vapor is confined in a cylinder glass cell, of which the length and diameter are both 25 mm. The cell is anti-relaxation coated and is kept at room temperature during the experiment. To increase the atomic number density, the vapor cell can be heated from 20 to 95 ${}^{\circ }$C [38]. For higher temperature, the coating on the inner wall of the cell becomes destroyed. The polarization of the transmitted probe laser from the cell is analyzed with a polarimeter and the signal is demodulated with a lock-in amplifier (Stanford Research Systems, SR865A, LIA). The demodulated signals are shown in Figure 1c, of which the in-phase component has a dispersive profile and a zero-crossing point when the modulation frequency is equal to the Larmor frequency, i.e., the frequency detuning is zero. The vapor cell, together with the optical components such as the wave plates, polarizers, and mirrors, are mounted in a 3D-printed structure, which has a size of 5×24×27 cm${}^{3}$ and is placed at the center of the coils [37].

The OPM sensor can be operated under two modes, i.e., the open-loop mode and the closed-loop mode. For the open-loop mode, we could tune the modulation frequency near the zero-crossing point (zero frequency detuning) and adopt the linear dependence between the voltage signal and frequency detuning to convert the measured voltage signal into magnetic field. For the closed-loop mode, the in-phase signal is used as an error signal to feedback control the modulation frequency to be equal to the Larmor frequency. The bandwidth of the OPM operated under the open-loop mode is determined by the linewidth of the resonance signal and is about 3 Hz in our system, see Figure 1c. Running the same OPM in the closed loop can extend the bandwidth to be larger than ∼1000 Hz, which is determined by the low-pass filter of the LIA [43] and is basically limited by the Larmor precession frequency [44]. A larger bandwidth, as will be shown later, is beneficial to improving the performance of active magnetic-field stabilization.

In our experiment, the OPM sensor is operated in the closed-loop mode. In the closed-loop mode, as the modulation frequency of the pump beam equals to the Larmor frequency of the bias magnetic field, we get the readout of the OPM through dividing the modulation frequency by the gyromagnetic ratio of Cs atoms. More specifically, the modulation signal in the OPM is generated with a function generator (Keysight 33520B), the modulation frequency of which is controlled with an inner voltage-controlled oscillator (VCO). We measure the input voltage signal of the VCO with a data acquisition card (National Instruments, USB6363) at a sampling rate of 40 kSa/s. Then we multiply the recorded voltage signal with the voltage to frequency transfer coefficient of the VCO, which gives the modulation frequency, and finally divide the modulation frequency by the gyromagnetic ratio to get the magnetic field readout.

The readout from the OPM sensor is used as an input signal for the feedback controller, which consists of PID modules (Proportion-Integration-Differentiation, Stanford Research Systems, SIM960) [45] and a VOC source (voltage-controlled current, Stanford Research Systems, LDC501). The PID module compares the measured result from the OPM with a reference and generates a signal, of which the amplitude is positively correlated to the difference between the measured result and the reference. Such a signal is used to control the output current of the VOC, which is connected to the inner layer of coils, and in turn, to compensate for the magnetic-field fluctuations. The basic procedures for actively stabilizing the magnetic field are summarized and illustrated as a block diagram shown in Figure 1d. The performance of the active magnetic-field stabilization is characterized by the noise rejection ratio, i.e., the ability to suppress the ambient magnetic-field noise. It is thus desirable to know how these factors shown in the block diagram affect this and how to improve the noise rejection ratio quantitatively.

We adopt the transfer function model in the control theory to calculate the noise rejection ratio of the active magnetic-field stabilization system [46,47]. The transfer function theoretically models a linear-time-invariant (LTI) system’s output for each possible input [48] and provides a direct way to quantitatively analyze the system’s characteristics, such as the stability and the frequency response. The active magnetic-field stabilization is a typical LTI system. The transfer function is normally calculated in the Laplace domain. For example, for continuous-time input signal x(t) and output signal y(t), the Laplace transform of the input and output is X(s)=L{x(t)} and Y(s)=L{y(t)}, respectively, where *s* is a complex parameter s=σ+iω, σ is a real number, i is the imaginary unit, ω is the angular frequency, and L{f(t)} is the Laplace transform for signal f(t). The transfer function is then calculated as H(s)=Y(s)/X(s).

The input signal of the active magnetic-field stabilization system is the environmental magnetic-field variation $B_{E}\left(s\right)$, see Figure 1d. The magnetic field used for compensating the magnetic-field variation is $B_{C}\left(s\right)$. The difference between $B_{E}\left(s\right)$ and $B_{C}\left(s\right)$ generates a noise-suppressed magnetic field $B_{S}\left(s\right)$, which is the input signal of the OPM sensor. FR(s) refers to the transfer function of the OPM sensor and is defined as the open-loop gain. The output of the OPM sensor is compared with a reference and produces an error signal for the feedback loop, which aims at zeroing the error signal. The transfer function of the feedback controller FB(s), together with that of the magnetic-field generator MG(s), gives the overall gain of the feedback loop. It should be noted that the parameters FR(s), FB(s), and MG(s) are nondimensionalized in the following analysis. This is valid given that FR(s)×FB(s)×MG(s) is dimensionless and is useful for extending the proposed model and analysis to magnetic-field stabilization using different kinds of magnetometers. In addition, since the sensor noise $B_{M}\left(s\right)$ also determines the noise level after field stabilization (not shown in Figure 1d), the stabilized magnetic field $B_{S}\left(s\right)$ is calculated as

(1) $$B_{S}\left(s\right)=B_{M}\left(s\right)+\frac{B_{E}\left(s\right)}{1+FR(s)\times FB(s)\times MG(s)},$$

indicating a rejection ratio of |1+FR(s)×FB(s)×MG(s)| for the environmental magnetic-field noise. Equation (1) provides a direct way to increase the noise rejection ratio by adjusting the corresponding parameters in the field stabilization system.

In practice, the noise from the feedback controller (the PID modules and the voltage-controlled current source) also more or less contributes to the noise level after field stabilization, but its contribution would not seriously affect the results if the feedback controller is set properly. According to Equation (1), to get a large noise rejection ratio, the PID modules should perform as amplifiers to amplify the readout of the OPM according to the PID process. During the amplification, the noise of the PID modules would be added to the output signal and reduce the signal-to-noise ratio, which eventually contributes to the system noise. Considering that the low-noise-amplifier technology is very mature, in general, this will not cause serious problems. As for the voltage-controlled-current source that drives the magnetic coil in our experiment, its noise could eventually be converted into the common-mode magnetic-field noise and be added to the environmental magnetic-field variation $B_{E}\left(s\right)$. Ideally, this common-mode magnetic-field noise can also be efficiently suppressed during the magnetic-field-stabilization process, as long as the current source together with the magnetic coil have sufficient resolution to respond to the required magnetic field changes, and the converted magnetic noise level does not exceed the noise suppression capability of the magnetic-field stabilization system. To reduce the requirement for the noise level of the current source, a convenient method is to reduce the current-to-magnetic-field coefficient of the magnetic coil, or to reduce the voltage-to-current coefficient of the voltage-controlled-current source, and then to increase the input of the voltage-controlled-current source by increasing the gain of the PID modules. This method, on the one hand, would help the current source and magnetic coil respond to smaller magnetic-field-changes requirements, and on the other hand, could reduce the converted magnetic-field noise from the current source.

The adjustment of the parameters FR(s), FB(s), and MG(s) also affects the status of stability of the system. An accurate and complete analysis on the system status is based on the Nyquist stability criterion by examining the roots of the equation 1+FR(s)×FB(s)×MG(s)=0 [49,50]. A stable system demands that the real parts of all the roots should be negative. Such a criterion is adequate enough to determine the status of the system’s stability, however, is not that straightforward to optimize the parameters by taking both of the noise rejection ratio and the stability into consideration. For a typical feedback controller shown in Figure 1d, the above criterion can be translated into a more direct one, i.e., |FR(s)×FB(s)×MG(s)| should be smaller than 1 at some specific frequency when the phase of [FR(s)×FB(s)×MG(s)] is $180^{\circ }$, corresponding to a positive feedback system. This is clear to understand, given that a positive feedback with a gain larger than 1 leads to self-perpetuation and/or amplification of the error signal. Based on this consideration, we construct a new parameter −1/[FB(s)×MG(s)]. Therefore, the intersection between the amplitudes of FR(s) and −1/[FB(s)×MG(s)] means that |FR(s)×FB(s)×MG(s)|=1, while the intersection between the phases of FR(s) and −1/[FB(s)×MG(s)] means that Phase$\left[FR\left(s\right)\times FB\left(s\right)\times MG\left(s\right)\right]=180^{\circ }$. For a stable system, these two intersections should be different (see Figures S1–S3 in the Supplementary Materials for detailed descriptions on the stability criterion).

**Figure 2:**
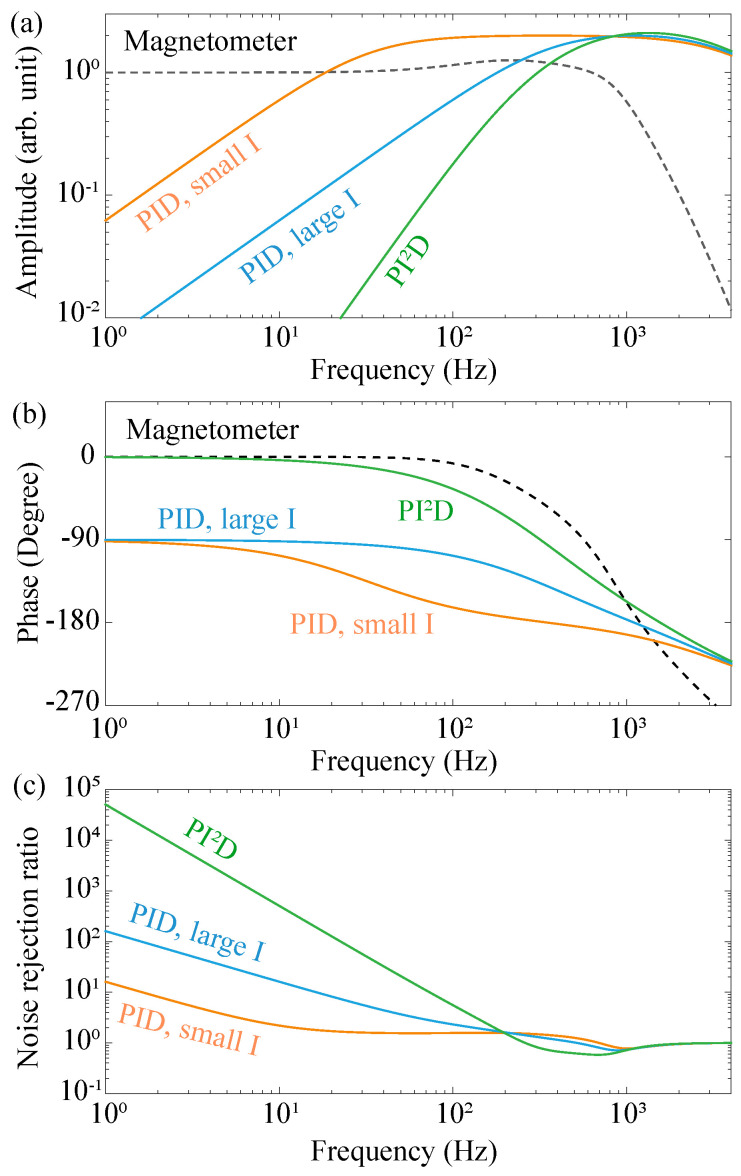
Optimization of the active magnetic-field stabilization system. (**a**) Amplitude–frequency responses of the feedback controller and the magnetometer. (**b**) Phase–frequency responses of the feedback controller and the magnetometer. (**c**) The calculated noise rejection ratios under different parameter settings of the feedback controller. The dashed black lines in (**a**,**b**) are the calculated amplitude- and phase- frequency responses of the magnetometer. The solid lines in (**a**,**b**) are the calculated amplitude- and phase- frequency responses of −1/[FB(s)×MG(s)]. Different colors of the lines indicate different parameters of the feedback controllers, i.e., orange (blue) for a PID module with a small (big) integration coefficient $K_{I}$, and green for a PI${}^{2}$D module.

Figure 2 summarizes the general idea of how to optimize the performance of an active magnetic-field stabilization system. We plot the amplitudes and phases of FR(s) and −1/[FB(s)×MG(s)] in Figure 2a,b, respectively. The horizontal axis is chosen as the frequency, or the imaginary part of the complex variable *s*, of which the real part is kept 0 since it is the frequency response of the different component that is measured experimentally. The dashed black lines are the calculated amplitude and phase of the frequency response of the magnetometer based on the experiment parameters. The magnetometer has a finite bandwidth, corresponding to the frequency where the signal amplitude is 3dB smaller than that of the low-frequency limit. The frequency response of −1/[FB(s)×MG(s)] is a combined effect from the feedback controller and the coil for field stabilization. The coil is taken as a first-order low-pass filter with a bandwidth of R/L, where *R* is the resistance and *L* is the inductance of the coil. In our system, the bandwidth of the coil is ∼7 MHz. The feedback controller is the PID module, of which the frequency response is determined by the coefficients of Proportion ($K_{P}$), Integration ($K_{I}$) and Differentiation ($K_{D}$) (see Figures S4–S6 in the Supplementary Materials for detailed forms of FB(s) and FR(s)).

The orange and blue lines in Figure 2a,b show the frequency responses of −1/[FB(s)×MG(s)] with different coefficients of the Integration, $K_{I}$. It is shown that a larger $K_{I}$ tends to shift the curve of the −1/[FB(s)×MG(s)] amplitude downward, and generates a larger noise rejection ratio, see Figure 2c. A detailed dependence of the frequency response of −1/[FB(s)×MG(s)] with different PID parameters ($K_{P}$, $K_{I}$ and $K_{D}$) is summarized in Figure S7 in the Supplementary Materials. Therefore, a general way for improving the noise rejection ratio is to adjust the parameters of the active magnetic-field stabilization system and increase the separation between the two curves shown in Figure 2a, i.e., the amplitude curves of −1/[FB(s)×MG(s)] and FR(s). During this process, the phase curve of −1/[FB(s)×MG(s)] is also shifted, which affects the status of stability of the system. As mentioned, the stability criterion demands that when the phase of [FR(s)×FB(s)×MG(s)] is $180^{\circ }$, |FR(s)×FB(s)×MG(s)| should be smaller than 1. This means that for a stable system, the intersection of the amplitude curves of FR(s) and −1/[FB(s)×MG(s)] should be at the left side of the intersection of the phase curves, see Figure 2a,b. A larger coefficient of the PID module reduces the separation in frequency between the two intersections, which sets a limit on the maximum noise rejection ratio. There are at least two ways to surpass this limit. One is to enhance the bandwidth of the magnetometer, which is equivalent to shift the black dashed line in Figure 2 to the right. An empirical conclusion is that, if the magnetometer bandwidth is increased by *N* times, the noise rejection ratio could be enhanced by the same amount (see the Supplementary Materials for detailed derivations). The other one is to increase the slope of the amplitude curve of −1/[FB(s)×MG(s)], for example, by adding an additional Integration to the PID module and constructing a PI${}^{2}$D module, see the green lines in Figure 2.

## 3. Results and Discussion

**Figure 3:**
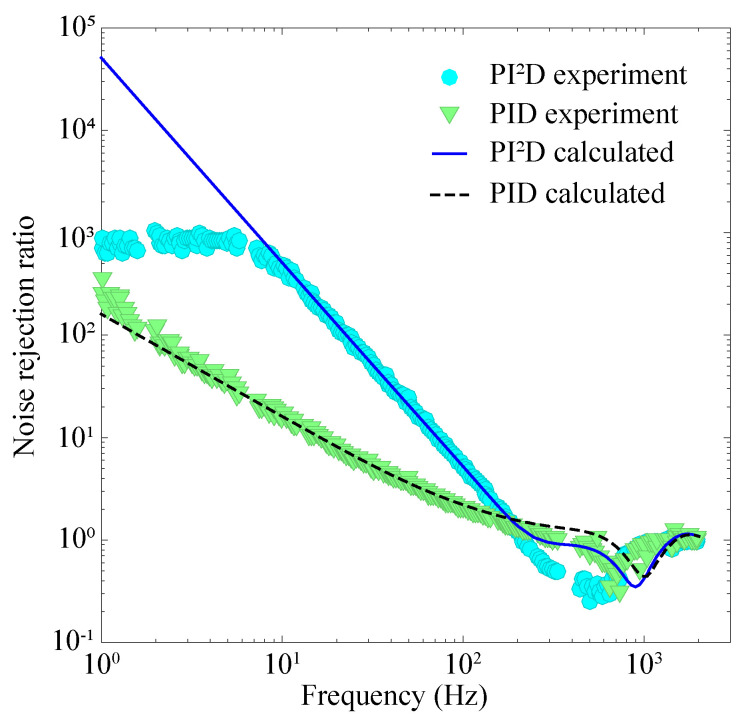
Noise rejection ratio of the active magnetic-field stabilization system. The green triangles and light blue circles show the experimentally measured noise rejection ratios using a PID module and a PI${}^{2}$D module, respectively. The calculated noise rejection ratios using a PID module and a PI${}^{2}$D module are shown by the black dashed line and dark blue solid line, respectively.

In order to characterize the noise rejection ratio of the active magnetic-field stabilization system, we place a second OPM in the system to monitor the magnetic-field noise before and after magnetic-field stabilization (OPM-M). We use a pair of Helmholtz coil to add a white magnetic-field noise to both of the two magnetometer sensors. More specifically, we use a signal generator (Keysight, 33520B) to produce a white electrical noise and feed it into the pair of Helmholtz coils along the vertical direction. This magnetic coil is connected in series with a 500 ohm resistance. The white magnetic-field noise has a bandwidth of 200 Hz and an amplitude of 58 pT/Hz${}^{1/2}$. To evaluate the experimentally achieved noise rejection ratios, we record the readouts of OPM-M under two different conditions, i.e., without and with magnetic-field stabilization, at a sampling rate of 40 kSa/s. Then we calculate the noise spectral density (NSD) of OPM-M readouts. After that, we divide the NSD without magnetic-field stabilization by the NSD with magnetic-field stabilization at the corresponding frequency, and the result means how many times the magnetic-field noise is suppressed at each frequency, or the noise rejection ratios. Figure 3 shows the experimentally achieved noise rejection ratios. The bias magnetic field is tuned around 20,000 nT. It is clear that the noise rejection ratio increases faster with the decreased frequency using a PI${}^{2}$D module compared with that using a PID module. The maximum measured noise rejection ratio (∼800) occurs around 10 Hz and has a constant value for lower frequencies for the PI${}^{2}$D module. The reason is that, for frequencies smaller than 10 Hz, the residual magnetic-field noise after stabilization is determined by the noise of the sensor for field stabilization (OPM-S). White magnetic-field noise with a larger amplitude is needed to measure the actual noise rejection ratio for lower frequencies.

We calculate the noise rejection ratio based on the proposed model in section II and compare the calculated results with the experimental results, see Figure 3. Based on Equation (1), the noise rejection ratio has the form as |1+FR(s)×FB(s)×MG(s)|. To calculate the noise rejection ratio based on Equation (1), we experimentally measure the frequency response of the magnetometer FR(s) together with that of the coil MG(s) by scanning the frequency of an oscillating signal added towards the magnetometer or the coil and recording the output signal, and we calculate the frequency response of the feedback controller FB(s) directly by using the expression of FB(s) and the experimental coefficients of $K_{P}$, $K_{I}$, and $K_{D}$ recorded from the device’s interface (SIM960). The calculated noise rejection ratios show good agreement with the experimental results for frequencies smaller than 200 Hz. For the PID module, a slight deviation between the theoretical and experimental results occurs for frequencies smaller than 3 Hz and is possibly due to the variations of the low-frequency magnetic-field noise of the environment and the magnetometer. A noise rejection ratio smaller than 1 is observed both experimentally and theoretically in the high-frequency region, which means that the field-stabilization system actually amplifies the magnetic-field noise. This happens when the phase of FR(s)×FB(s)×MG(s) is around $180^{\circ }$ and the modulus of FR(s)×FB(s)×MG(s) is smaller than 1, see Figure 2. We also notice that, for both of the PID and PI${}^{2}$D modules, the deviation between the theoretical and experimental results is larger in the high-frequency region. Such a deviation may come from the phase shifts of the devices that are not considered in the current model, such as the attenuator, and could be reduced by adding additional phase-shift contributions from these devices into the calculations. In general, Figure 3 shows that the current model is precise enough to provide a quantitative approach in optimizing and calculating the noise rejection ratio in the low-frequency region. Given the slope of the curve, the noise rejection ratio is higher for lower frequencies, indicating a noise rejection ratio of ∼10${}^{5}$ at 1 Hz by using the PI${}^{2}$D module. A larger noise rejection ratio means that the system has a better performance in reducing the ambient magnetic-field noise, however, does not guarantee a lower magnetic-field noise level after field stabilization. The noise level after magnetic-field stabilization is also determined by the noise level of the magnetometer, see Equation (1), and the homogeneity of the magnetic fields generated from the coils.

**Figure 4:**
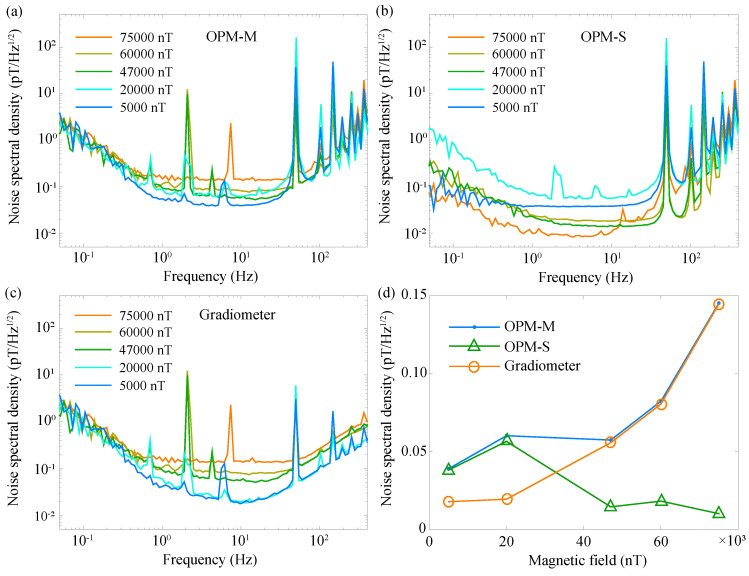
Noise spectral density (NSD) measured with the OPM sensors after active magnetic-field stabilization under different strength of bias magnetic field. (**a**) The NSD of OPM-M sensor, i.e., the magnetometer used for monitoring the magnetic-field noise after magnetic-field stabilization. (**b**) The NSD of OPM-S, i.e., the magnetometer used for magnetic-field stabilization. (**c**) The NSD of the gradiometer. (**d**) The NSD of OPM-M, OPM-S, and the gradiometer at 10 Hz under different strength of bias magnetic field.

We use both of the two magnetometers to record the magnetic-field noise after field stabilization. The bias magnetic-field strength is adjusted to be some discrete values from 5000 nT to 75,000 nT. The recorded noise level of the two magnetometers, including a subtraction between them, are shown in Figure 4. Figure 4a shows the measured noise level of OPM-M, the magnetometer solely for measuring the magnetic-field noise. A noise floor of 40 fT/Hz${}^{1/2}$ around 10 Hz is achieved under a bias field of 5000 nT. The measured noise floor increases with the strength of the bias magnetic field, given that the nonlinear Zeeman effect tends to broaden the linewidth of the resonance signal and degrade the sensitivity of the magnetometer. For frequencies larger than 20 Hz, the noise level increases with frequency since the closed-loop operation only enhances the bandwidth of the magnetometer while it does not improve the signal-to-noise ratio. For frequencies smaller than 1 Hz, the noise level is higher for lower frequencies. Besides the 1/f noise of magnetometer, a large fraction of the noise is from the magnetic-field gradient noise, which means that the magnetic-field noise is different for the two magnetometers. The data shown in Figure 4a are all taken during the daytime. Typically, the environment is magnetically-quieter at midnight than in the daytime and has a lower magnetic-field gradient noise. We find that the measured noise level in the midnight for frequencies smaller than 1 Hz is smaller than that shown in Figure 4a (see Figure S8 in the Supplementary Materials for a detailed comparison between magnetic-field noise level recorded in the daytime and midnight).

The noise level of the magnetometer for field stabilization (OPM-S) shows quite different characteristics, see Figure 4b. Generally, the noise level is smaller for larger bias magnetic fields and becomes 9 fT/Hz${}^{2}$ when the bias field strength is 75,000 nT. Such a phenomenon is quite interesting since it proves that, with the active magnetic-field stabilization system, some of the magnetic-like-noise of the magnetometer for field stabilization, for example, the noise originating from the vector light shift [51], are taken as the magnetic-field noise and is actively stabilized. Since these magnetic-like-noises are not common-mode noises for the two magnetometers, the magnetic field used for compensating the magnetic-like-noise directly added into the measured results of OPM-M. Therefore, the measured noise level in Figure 4b are all smaller than those shown in Figure 4a. The magnetic-field noise along the horizontal directions adds in quadrature to the dominant vertical bias magnetic field, i.e., $B_{x,y}^{2}/B_{0}$, where $B_{x,y}$ is the magnetic-field noise along the horizontal directions and $B_{0}$ is the bias magnetic field. For a smaller bias magnetic field, the effect from the magnetic-field noise along the horizontal directions becomes larger. This explains why the noise level shown in Figure 4b is higher for a smaller strength of the bias magnetic field.

The magnetic-field noise along the horizontal directions are the common-mode noise for the two magnetometers. Thus, such noise could be eliminated by subtracting the measured results from the two magnetometers, see Figure 4c. The noise level shown in Figure 4c is almost identical to those shown in Figure 4a, except for a lower noise floor for smaller magnetic fields, i.e., 5000 nT and 20,000 nT, under which the effect from the common-mode horizontal magnetic-field noise is larger and is suppressed through subtracting the measured results from the two magnetometers. The measured noise levels of the two magnetometers and the gradiometer at a certain frequency (10 Hz) with different magnetic fields are summarized in Figure 4d. In summary, the Earth’s field along the vertical direction has several effects. The first one is that the noise of the vertical Earth’s field directly determines the ambient magnetic noise floor, as the OPMs are most sensitive to the magnetic signal along the vertical direction in our experiment. The second effect comes from the gradient noise of the Earth’s field, which degrades the performance of the magnetic-field stabilization, as the variance of the magnetic gradient noise is not a common-mode noise and cannot be eliminated by the magnetic-field stabilization in our experiment. To solve this problem, we need to introduce more magnetometers for measuring the magnetic gradient and some gradient magnetic coils for actively compensating the gradient noise. The third effect comes from the nonlinear Zeeman effect, which is more obvious for the larger magnetic field. The non-negligible nonlinear Zeeman effect under the Earth’s field leads to the broadening of the magnetic resonance signal and a decrease of the signal-to-noise ratio, and eventually decreases the sensitivity of the OPM. The fourth effect is that a large vertical magnetic field helps to decrease the effect from the magnetic-field noise along the horizontal directions on the magnetic-field stabilization in our experiment.

## 4. Conclusions and Outlook

In this work, we present a model to calculate the noise rejection ratio of the active magnetic-field stabilization using atomic magnetometers. We show how to improve the performance of the field stabilization both experimentally and theoretically through using atomic magnetometers. As is shown in Equation (1), the sensitivity of the magnetometer and the ability to suppress ambient magnetic noise are the key factors to determine the noise floor after magnetic-field stabilization. To improve the sensitivity of our OPMs, we use anti-relaxation coated vapor cells with narrow magnetic resonance, which makes the atomic ensemble more sensitive to magnetic signals, and suppress technical noises induced by the fluctuations of laser power and frequency. To improve the noise rejection ratio, we add an additional Integration to the PID module and construct a PI${}^{2}$D module. With this effort, we experimentally demonstrate a noise rejection ratio of larger than 800 for frequencies smaller than 10 Hz and a noise floor of about 40 fT/Hz${}^{1/2}$ in an unshielded Earth’s field. The proposed model can be extended to active magnetic-field stabilization using other kinds of magnetometers, such as SQUIDs, fluxgate, or inductive coils. Our work is important for highly-sensitive detections of magnetic-field signals in an unshielded environment. For example, recording the magnetic field signals generated from human brain activities in an unshielded environment is important for the clinical applications and reducing the cost of an MEG system. Our work can also be used in fundamental physics experiments, for example, searches for permanent EDM and axionlike dark matter. For these experiments, regardless of the usage of comagnetometer, which is for suppressing the magnetic-field noise, an environment with lower magnetic-field noise is beneficial for further reducing the systematic errors related to magnetic-field variations. Furthermore, a magnetic-field noise level of about tens of fT/Hz${}^{1/2}$ and a wide range of operational magnetic field covering Earth’s magnetic field is attractive for ultralow-field nuclear magnetic resonance [52,53] and for recording bio-magnetic signals from human brain activities [54,55].

Besides these potential applications, there are still some further improvements to be implemented for the current field-stabilization system. For example, we could broaden the bandwidth of the magnetometer for field stabilization even further to achieve a larger noise rejection ratio. The current bandwidth of the magnetometer is ∼1 kHz, which is determined by the time constant of the lock-in amplifier. In reality, the bandwidth of the atomic magnetometer is fundamentally determined by the Larmor precession frequency, which is proportional to the strength of the bias magnetic field, for example, ∼100 kHz for the cesium atoms under Earth’s field. If the magnetometer is operated in the instantaneous-phase retrieval mode, the bandwidth can even go beyond the Larmor frequency [44]. As a result, enhancing the noise rejection ratio through increasing the bandwidth of the atomic magnetometer is also possible in a small bias field. In addition, since a better performance in reducing the low-frequency magnetic-field noise is achieved by adding an additional Integrator, along this way, it is also possible to further improve the noise rejection ratio by introducing more Integrators. However, since adding an additional Integrator affects the stability of the field-stabilization system, there exists a limit for the maximum achievable noise rejection ratio. Furthermore, the proposed method in this paper can also be adapted and used to stabilize the magnetic-field gradient noise, together with using more magnetometers to monitor, and in turn, to stabilize the spatial distribution of the magnetic fields [56]. This is beneficial for experiments that operate in a more magnetically-noisy environment, for example, a laboratory situated in the center of the city, which is surrounded with intense magnetic disturbances originating from vehicles, elevators and some other moving magnetic substances.

## Supplementary Materials

The following are available online at https://www.mdpi.com/1424-8220/20/15/4241/s1, Figure S1: Stability analysis based on the Nyquist diagram and Bode diagrams when the open loop transfer function G(s) does not have any pole on the imaginary axis, Figure S2: Stability analysis based on the Nyquist diagram and Bode diagrams when the open loop transfer function G(s) has a pole on the imaginary axis, Figure S3: Stability analysis based on Nyquist diagrams and Bode diagrams when the open loop transfer function G(s) has a double pole on the imaginary axis, Figure S4: A typical Bode diagram of the PID controller, Figure S5: A typical Bode diagram of the PI${}^{2}$D controller, Figure S6: Block diagram of the closed-loop operation of the AM NMOR magnetometer, Figure S7: The influence of the PID coefficients on the magnetic-field stabilization system, Figure S8: Noise spectral density at different time.
