# Supplementary material for: Active Magnetic-Field Stabilization with Atomic Magnetometer
Source: Sensors (Basel). 2020 Jul 30;20(15):4241. doi: 10.3390/s20154241 (PMC7435849; doi:10.3390/s20154241)
Supplement: Supplementary file 1 [file sensors-20-04241-s001.zip › sensors-856128-Supplementary/SupplementaryMaterial.pdf]

Supplementary Material

(Dated: June 20, 2020)

SECTION S1. TRANSFER FUNCTIONS AND STABILITY CRITERIA OF LINEAR SYSTEMS

section S1.1 The general transfer function of linear systems

The magnetic-field stabilization system can be modelled as the single-input-single-output (SISO) linear-time-invariant (LTI) system, whose input $In(t)$ is the environmental magnetic-field variation and output $Out(t)$ is the noise-suppressed magnetic field. The word "linear" means that for every arbitrary inputs $In_1(t)$ and $In_2(t)$, if the corresponding outputs of the system are $Out_1(t)$ and $Out_2(t)$ respectively, then for the input $\alpha \times In_1(t) + \beta \times In_2(t)$ the corresponding output is $\alpha \times Out_1(t) + \beta \times Out_2(t)$, in which α and β are arbitrary numbers. As an example, proportions, integrators and differentiators are common linear systems. Generally speaking, a SISO LTI system can be described by a differential equation as follows [1]:

$$\begin{aligned} &C_0 \times Out(t) + \sum_{k=1}^{m} C_k \frac{\mathrm{d}^k Out(t)}{\mathrm{d}t^k} \\ =&D_0 \times In(t) + \sum_{l=1}^{n} D_l \frac{\mathrm{d}^l In(t)}{\mathrm{d}t^l}, \end{aligned} \tag{S1}$$

and typically, we assume that the input signal starts from time $t = 0$. Note that there is no non-zero constant term in this equation, because a non-zero constant term will cause the equation to lose its "linear" characteristic. For each input, we can get the corresponding output of the system by solving this differential equation.

Compared with solving the differential equation eq. (S1) in the time-domain, an alternative and many times more convenient method is analyzing it in the frequency domain. In the frequency domain, eq. (S1) is transferred to an algebraic equation, which can be solved easier. To do so, we need to Laplace transform the eq. (S1). The Laplace transformation of a function $x(t)$ is the function $\mathcal{L}\left[x(t)\right]$ defined by

$$\mathcal{L}\left[x(t)\right] = \int_0^{\infty} x(t) e^{-st} \mathrm{d}t. \tag{S2}$$

In this equation, $s = \sigma + \mathrm{i}\omega$ is the independent variable in the Laplace domain, in which σ is a real number, i is the imaginary unit, and ω is the angular frequency of the signal and is also a real number. To transform eq. (S1) into the Laplace domain, an useful properties of the Laplace transformation is

$$\mathcal{L}\left[\frac{\mathrm{d}x(t)}{\mathrm{d}t}\right] = s \times \mathcal{L}\left[x(t)\right] - x(0^-). \tag{S3}$$

Typically, the initial value $x(0^-)$, or the value of the function just before time instant $t = 0$, is assumed to be zero. Even if the function $x(t)$ jumps at $t = 0$, as long as the initial values $x(0^-)$ and all of its time derivatives are zeros, the time differentiation is still reduced to

$$\mathcal{L}\left[\frac{\mathrm{d}^j x(t)}{\mathrm{d}t^j}\right] = s^j \times \mathcal{L}\left[x(t)\right], \tag{S4}$$

in which j is an arbitrary positive integer. With the help of eq. (S4), if we apply the Laplace transformation to both sides of eq. (S1), we will find that

$$\mathcal{L}\left[Out(t)\right] \times \sum_{k=0}^{m} C_k \times s^k = \mathcal{L}\left[In(t)\right] \times \sum_{l=0}^{n} D_l \times s^l. \tag{S5}$$

or

$$\mathcal{L}\left[Out(t)\right] = \frac{\sum_{l=0}^{n} D_l \times s^l}{\sum_{k=0}^{m} C_k \times s^k} \mathcal{L}\left[In(t)\right]. \tag{S6}$$

As a result, we can define the transfer function as

$$H(s) = \frac{\mathcal{L}\left[Out(t)\right]}{\mathcal{L}\left[In(t)\right]} = \frac{\sum_{l=0}^{n} D_l \times s^l}{\sum_{k=0}^{m} C_k \times s^k}, \tag{S7}$$

which describes the relationship between the input and output signals of the LTI system in the Laplace domain. The transfer function can also be written in the zero-pole-gain form:

$$H(s) = A \frac{\prod_{l=1}^{n}(s - z_l)}{\prod_{k=1}^{m}(s - p_k)}, \tag{S8}$$

in which the z_l represents the roots of the numerator, or the zeros of the transfer function, and p_k represents the roots of the denominator, or the poles of the transfer function. Besides, n should not be larger than m for a physically realizable system, otherwise the output signal will not remain finite for finite changes of the input signal [1].

section S1.2 The general stability criterion for linear systems

For a SISO LTI system, the stability means that when a disturbance is imposed on the input and drives the system away from its equilibrium, the system is able to recover from input disturbance and get back into the equilibrium state again [2]. To be a stable system, each pole p_l of its transfer function $H(s)$, see eq. (S8), should have a negative real part. A rigorous deduction of this criterion can be found in [3]. Here we use a simple example to illustrate the necessity of this criterion.

Assuming that there is a jump in the input at time instant $t = 0$, if the system is stable, the system output will eventually return to an equilibrium. Whether the system would return to an equilibrium can be easily evaluated in the time domain. To get the time-domain output $Out(t)$, according to the definition of the transfer function, see eq. (S7), we could first obtain the Laplace transformation of the output signal, or $\mathcal{L}[Out(t)]$, through multiplying the Laplace transformation of the input signal, or $\mathcal{L}[In(t)]$, and the transfer function $H(s)$ of the system, and then apply the inverse Laplace transformation to the multiplication result.

In this case, the jump in the input signal can be modelled by the step function $1(t)$, which is 0 before $t = 0$ and jumps to 1 from time instant $t = 0$. The Laplace transformation of $1(t)$ is

$$\mathcal{L}[1(t)] = \frac{1}{s}, \tag{S9}$$

so that the Laplace transformation of output signal $Out(t)$ is

$$\mathcal{L}[Out(t)] = \mathcal{L}[1(t)] \times H(s) = \frac{1}{s} \times H(s). \tag{S10}$$

Then we could get $Out(t)$ through applying the inverse Laplace transformation to eq. (S10).

However, commonly it is very complex to directly apply the inverse Laplace transformation to a function. An alternative and relatively simpler way is making use of the linearity property of the inverse Laplace transformation, which makes it possible to get the inverse Laplace transformation through decomposing the Laplace transformation $\mathcal{L}[Out(t)]$ into some known transformations of functions that can be obtained from a table. Linearity of the inverse Laplace transformation means that if we have two functions $X_1(s)$ and $X_2(s)$, and their inverse Laplace transformations are $\mathcal{L}^{-1}[X_1(s)]$ and $\mathcal{L}^{-1}[X_2(s)]$ respectively, then the inverse Laplace transformation of any arbitrary linear combination of these two functions, or $\mathcal{L}^{-1}[\alpha X_1(s) + \beta X_2(s)]$, is the same linear combination of their inverse Laplace transformations, or $\alpha\mathcal{L}^{-1}[X_1(s)] + \beta\mathcal{L}^{-1}[X_2(s)]$, in which α and β are arbitrary numbers. As a result, we can decompose $\mathcal{L}[Out(t)]$ into the sum of some simpler parts and $Out(t)$ is the sum of the inverse Laplace transformation of each part.

To decompose $\mathcal{L}[Out(t)]$, we can rearrange the transfer function $H(s)$ in eq. (S8) to the partial fractional form (supposing there is no repeated pole, or any two poles are different):

$$H(s) = E_0 + \sum_{k=1}^{m} \frac{E_k}{s - p_k}, \tag{S11}$$

in which p_k is the poles and E_k is the residuals of the transfer function [1]. As a result, according to eq. (S10) the Laplace transformation of the output signal is

$$\mathcal{L}[Out(t)] = \frac{E_0}{s} + \sum_{k=1}^{m} \frac{E_k}{s(s - p_k)}. \tag{S12}$$

For the cases where no pole equals zero, as

$$\mathcal{L}\left[e^{at} - 1\right] = \frac{a}{s(s-a)}, \tag{S13}$$

the output in the time domain would be

$$Out(t) = E_0 \times 1(t) + \sum_{k=1}^{m} E_k \times \frac{e^{p_k t} - 1}{p_k}. \tag{S14}$$

For each pole p_k, if it has a negative real part, or $\mathrm{Re}(p_k) < 0$, its contribution to $Out(t)$, or $E_k \times (e^{p_k t} - 1)/p_k$, will get back into the equilibrium state $-E_k/p_k$ eventually. But if any pole p_k has a non-negative real part, its contribution to $Out(t)$ will contain an exponentially increased oscillating component (for $\mathrm{Re}(p_k) > 0$) or a sinusoidal oscillating component (for $\mathrm{Re}(p_k) = 0$), so that the system is unstable. For some other cases where a pole equals 0, for example $p_1 = 0$, the Laplace transformation of the output signal is

$$\mathcal{L}[Out(t)] = \frac{E_0}{s} + \frac{E_1}{s^2} + \sum_{k=2}^{m} \frac{E_k}{s(s - p_k)}. \tag{S15}$$

As

$$\mathcal{L}[t] = \frac{1}{s^2}, \tag{S16}$$

the output in the time domain would be

$$Out(t) = E_0 \times 1(t) + E_1 \times t + \sum_{k=2}^{m} E_k \times \frac{e^{p_k t} - 1}{p_k}. \tag{S17}$$

As a result, there is a component that increases with time t in the output, and the system is also unstable. As a conclusion, only when each pole has a negative real part, or $\mathrm{Re}(p_k) < 0$ for every k, the system is stable. For the cases with repeated poles, we can get the same conclusion in a similar way.

section S1.3 Stability criteria based on the Nyquist diagrams or Bode diagrams

According to the general stability criterion of LTI systems, we can evaluate the stability of a system through analytically or numerically finding out all the poles of its transfer function $H(s)$ and then checking whether each of them has a negative real part. But this method is not intuitive about how to change the parameters of the system to optimize its dynamical performance, especially in the case of instability. Comparatively, the stability criteria based on the Nyquist diagrams or Bode diagrams provides a more intuitive method to optimize the system performance.

Taking the magnetic-field stabilization system shown in Fig. 1(d) in the main text as an example, it transfer function is

$$H(s) = \frac{B_{\mathrm{S}}(s)}{B_{\mathrm{E}}(s)} = \frac{1}{1 + FR(s) \times FB(S) \times MG(s)}, \tag{S18}$$

in which $FR(s) \times FB(S) \times MG(s)$ is the open-loop transfer function. In the following analysis we mark $FR(s) \times FB(S) \times MG(s)$ as $G(s)$. The poles of $H(s)$ are actually the zeros of $1 + G(s)$, so that we can evaluate the stability of the closed-loop system through counting the number of zeros of $1 + G(s)$ that locate on the right half-plane. According to the Cauchy argument principle, if s moves on the s plane along a closed curve that does not pass through any zeros and poles of $1+G(s)$, then the number of times the mapping by $1 + G(s)$ of the closed curve encircles the origin clockwise is the difference of the number of zeros and the number of poles of $1 + G(s)$ inside the s curve [4]. As a result, if s moves along a closed curve that just encloses the entire right half-plane, and if $1+G(s)$ has Z zeros and P poles on the right half-plane, then the number of times the mapping by $1+G(s)$ of the closed curve winds around the origin clockwise is

$$R = Z - P. \tag{S19}$$

As long as we know R and P, we would be able to figure out Z, which should be 0 for a stable system. In practice, because the number of times the mapping by $1 + G(s)$ encircles the origin equals the number of times the mapping by $G(s)$ encircles the point (-1, 0), and because $1+G(s)$ and $G(s)$ share the same poles, we use the mapping by $G(s)$ of the considered closed curve, which is known as the Nyquist diagram of $G(s)$, to evaluate the stability of the system. Such a stability criterion is called the Nyquist criterion [2].

As an example, we use the Nyquist criterion to evaluate the stability of a system with $G_1(s) = 8/[(1 + s)^6]$, and the results are shown in fig. S1. This $G_1(s)$ function has no pole on the imaginary axis, as a result the closed curve of s can be chosen as that shown in fig. S1(a). The closed curve is divided into 3 parts:

1) The first part is on the imaginary axis, from $\omega = 0$ to $\omega \to +\infty$, indicated by the black solid line.

2) The second part is a half-circle on the right half-plane, whose radius r tends to be infinite, indicated by the red dashed line.

3) The third part is also on the imaginary axis, from $\omega \to -\infty$ to $\omega = 0$, indicated by the blue dashed-dotted line.

These three parts together form a closed curve that totally encloses the right half-plane. The Nyquist diagram is also divided into 3 parts, corresponding to the mapping of the 3 segments of the closed curve on the s plane (each curve segment to be mapped shares the same color and line shape with its mapping by $G_1(s)$):

1) The first part starts from a point on the positive half of the real axis, corresponding to $G_1(0)$. As ω increases, it moves clockwise along the black line and passes the negative half of the real axis at point $(-a, 0)$ and eventually approaches the origin as $\omega \to +\infty$.

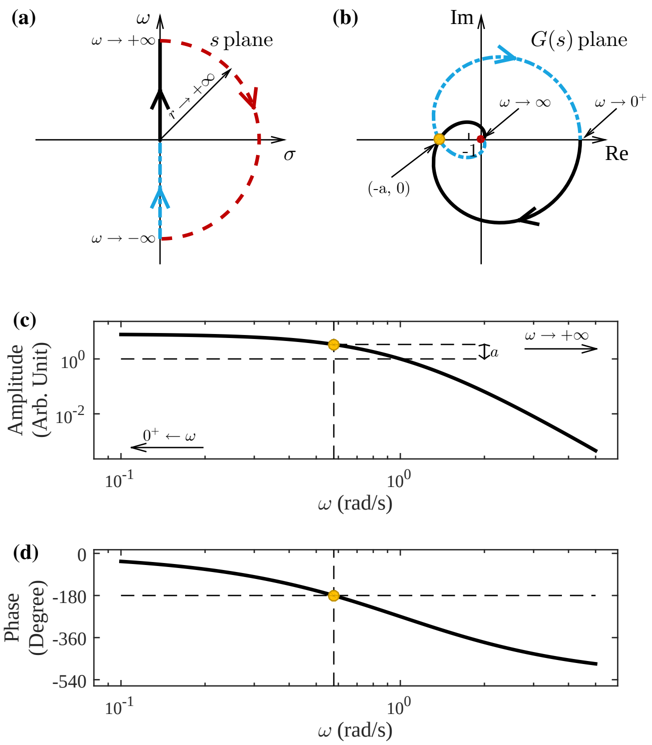

FIG. S1. **Stability analysis based on the Nyquist diagram and Bode diagrams when the open loop transfer function $G(s)$ does not have any pole on the imaginary axis.** (a) The closed curve to be mapped. This curve is divided into three segments, which are indicated by the black solid line, the red dashed line and the blue dashed-dotted line respectively. (b) The Nyquist diagram of $G_1(s) = 8/[(1+s)^6]$, i.e. the mapping by $G_1(s)$ of the closed curve on the s plane shown in (a). Each segment of the closed curve in (a) shares the same color and line shape with its mapping shown in (b). (c) and (d) are the Bode amplitude-frequency diagram and the Bode phase-frequency diagram of $G_1(s)$ respectively.

2) The second part is fixed at the origin, corresponding to the mapping of the half-circle on the s plane with infinite radius.

3) The third part is the mirror image of the first part related to the real axis.

As the point that the mapping by $G_1(s)$ goes across the negative half of the real axis, or point $(-a, 0)$, is at the left of the point (-1, 0), the point (-1, 0) is encircled by the mapping for two times, i.e. $R = 2$. Because $1 + G(s)$ does not have any pole on the right half-plane, or $P = 0$, the number of zeors of $1 + G(s)$ on the right half-plane is $Z = R - P = R - 0 = 2$. As a result, $1 + G(s)$ has two zeros on the right half-plane, which means that the corresponding closed-loop transfer function $H(s)$ has two poles on the right half-plane and that the system is unstable.

The Nyquist criterion provides us an intuitive suggestion on how to optimize the feedback to make the system

stable. If we move the $(-a,\, 0)$ point to the right, like through decreasing the overall gain of $G_1(s)$, until it coincidences with (-1, 0), then $1 + G(s)$ has zeros on the imaginary axis, and the system is at the stability limit. If the $(-a,\, 0)$ point is moved further that it is located at the right of (-1, 0), then the mapping by $G_1(s)$ would not encircle the point (-1, 0), and as a result $Z = 0$ and the system is stable.

The stability of the system can also be evaluated based on the Bode diagram [2]. Compared with the Nyquist diagram, the Bode diagram provides us additional information about the frequency dependence of the transfer function, which is helpful for the parameter optimization of the feedback system. Though the Nyquist diagram shown in fig. S1(b) is composed of three parts, we can merely use its first part to evaluate the stability of the system, because the second part is a fixed point at the origin, and the third parts is only the mirror image of the first part. The amplitude and phase of the first part of the mapping by $G_1(s)$ can be plotted separately as a function of ω in fig. S1(c) and (d), which are known as the Bode amplitude-frequency diagram and the Bode phase-frequency diagram respectively. According to the Nyquist stability criterion, the system is stable only when the $(-a,\, 0)$ point, the point where the mapping by $G_1(s)$ in the Nyquist diagram goes across the negative half of the real axis, is at the left of the point (-1, 0). **The equivalent criterion in the Bode diagram is that the amplitude of $G_1(s)$ is smaller than 1 at the frequency where the phase of $G_1(s)$ is $-180°$.** The case shown in fig. S1(c) has a amplitude larger than 1 at the frequency where phase of $G_1(s)$ is $-180°$, so that the system is unstable, just the same as what we get from the Nyquist diagram fig. S1(b). This Bode diagram based stability criterion is exactly what we use in the main text.

There are also cases that $G(s)$ has some poles in the imaginary axis. Thus the original closed curve on the s plane should be modified to get around the given point with a half-circle of infinitesimal radius δ. This situation is common when integrations are involved in the feedback. For example, the $G(s)$ of the magnetic-field stabilization system with a PID module has a single pole at the origin, and the $G(s)$ of the stabilization system with a PI^{2}D module has a double pole at the origin.

An example that $G(s)$ has a single pole at the origin is shown in fig. S2, in which $G_2(s) = 150/[s \times (s+1-5\mathrm{i}) \times (s+1+5\mathrm{i})]$ is used. As shown in fig. S2(a), the closed curve in the s plane goes along two quarter-circle of infinitesimal radius δ successively, indicated by the green dashed line and the orange dotted line respectively, to get around the origin from the right. As a result, the pole on the origin is not enclosed by the closed curve and can be considered as a pole in the left half-plane, which is not included in P. The mapping by $G_2(s)$ of the orange quarter-circle of infinitesimal radius δ starts from the positive half of the real axis and goes clockwise along a quarter-circle with infinite radius, indicated by the orange dotted line in fig. S2(b), and ends at the negative half of the imaginary axis. The mapping of the green quarter-circle with infinitesimal radius δ is the mirror image of the mapping of the orange quarter-circle related to the real axis, and is indicated by the green dashed line in fig. S2(b). The mapping by $G_2(s)$ shown in fig. S2(b) encircles the (-1, 0) point clockwise for two times, which indicates that $R = 2$. Because $1 + G_2(s)$ does not have any pole on the right half-plane, or $P = 0$, the number of zeros of $1 + G_2(s)$ on the right half-plane is $Z = R - P = R - 0 = 2$. As a result, the overall transfer function $H(s)$ has two poles on the right half-plane and the system is unstable. When it comes to drawing the Bode diagram, strictly speaking, we should consider both the black solid line and the orange dotted line of the Nyquist diagram in fig. S2(b), as both of these two curves are nontrivial while the blue dashed-dotted line and the green dashed line are the mirror images of them.

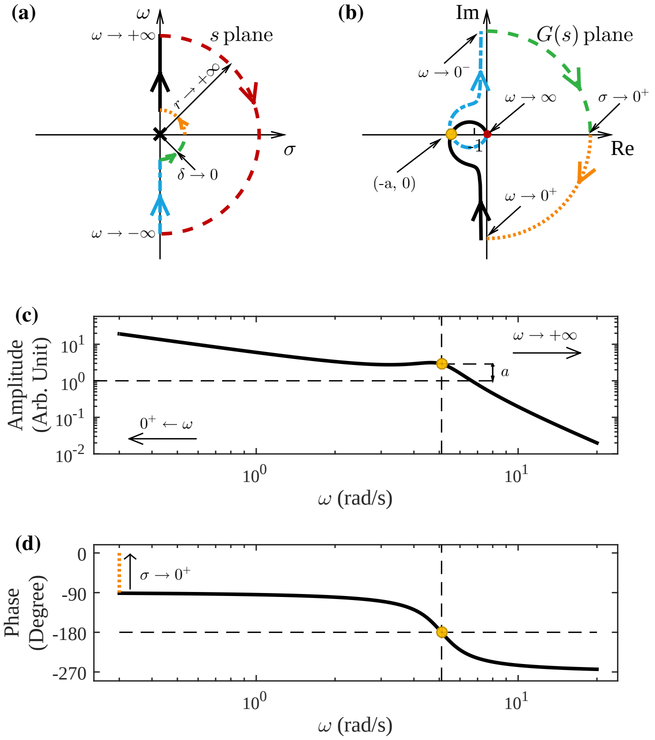

FIG. S2. **Stability analysis based on the Nyquist diagram and Bode diagrams when the open loop transfer function $G(s)$ has a pole on the imaginary axis.** (a) The closed curve to be mapped when $G(s)$ has a pole on the imaginary axis. This curve is divided to five segments, which are indicated by the orange dotted line, the black solid line, the red dashed line, the blue dashed-dotted line and the green dashed line respectively. (b) The Nyquist diagram of $G_2(s) = 150/[s \times (s+1-5\mathrm{i}) \times (s+1+5\mathrm{i})]$, i.e. the mapping by $G_2(s)$ of the closed curve in (a). Each segment of the closed curve in (a) shares the same color with its mapping in (b). (c) and (d) are the Bode amplitude-frequency diagram and the Bode phase-frequency diagram of $G_2(s)$ respectively.

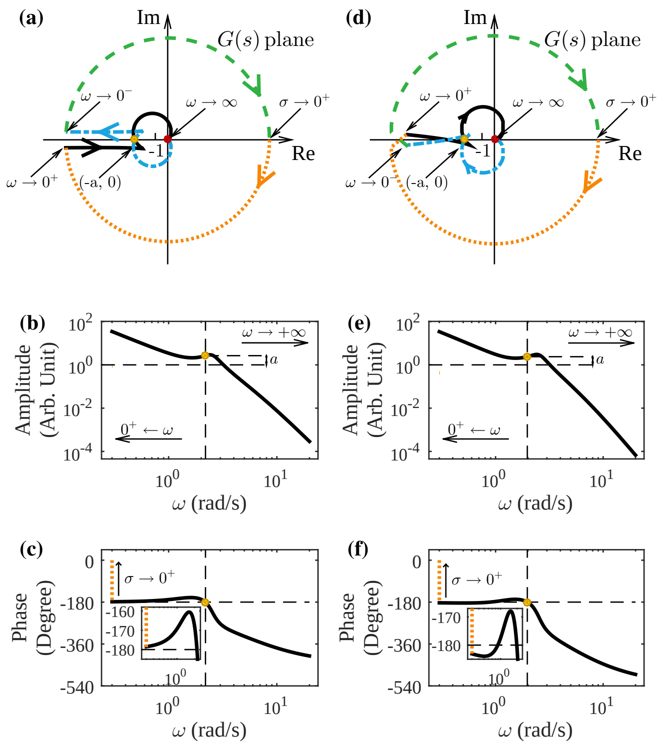

FIG. S3. **Stability analysis based on Nyquist diagrams and Bode diagrams when the open loop transfer function $G(s)$ has a double pole on the imaginary axis.** (a), (b) and (c) are the Nyquist diagram, the Bode amplitude-frequency diagram and the Bode phase-frequency diagram of $G_3(s) = (20/s^2 + 16/s + 8)/[(0.2 \times s + 1)^3 \times (s + 0.4 - 2.5\mathrm{i}) \times (s + 0.4 + 2.5\mathrm{i})]$ respectively. (d), (e) and (f) are the Nyquist diagram, the Bode amplitude-frequency diagram and the Bode phase-frequency diagram of $G_4(s) = (20/s^2+16/s+11)/[(0.22 \times s+1)^4 \times (s+0.4-2.5\mathrm{i}) \times (s+0.4+2.5\mathrm{i})]$ respectively. The insets in (c) and (f) show the details about the Bode phase-frequency diagram of $G_3(s)$ and $G_4(s)$ in the low frequency range respectively.

The orange quarter-circle with infinite radius in fig. S2(b) corresponds a point at $\omega \to 0^+$ with infinite hight in the amplitude-frequency diagram, which is not shown in fig. S2(c), and its contribution to the phase-frequency diagram is a vertical line at $\omega \to 0^+$ with hight from 0° to $-90°$, indicated by the orange dotted line in fig. S2(d). According to the Bode diagram based stability criterion, this system is also unstable because the amplitude at ω where phase is $-180°$ is larger than 1. Besides, as the orange vertical line in fig. S2(d) does not go across the $-180°$ line, it can be omitted, just as what we do in the main text.

For the case that $G(s)$ has a double pole at the origin, in other words, there are two poles located in the origin, the closed curves to be mapped are the same as that shown in fig. S2(a), but their Nyquist diagrams and Bode diagrams have some new characteristics. An example with $G_3(s) = (20/s^2+16/s+8)/[(0.2\times s+1)^3 \times (s+0.4-2.5\mathrm{i}) \times (s+0.4+2.5\mathrm{i})]$ is shown in fig. S3(a), (b) and (c). One difference introduced by the double pole is that the mapping by $G_3(s)$ of the orange quarter-circle with infinitesimal radius in fig. S2(a) is a half-circle with infinite radius, indicated by the orange dotted line in fig. S3(b), rather than the the quarter-circle with infinite radius in the single pole case shown in fig. S2(b). As a result, the contribution from this mapping to the phase-frequency diagram is a vertical line at $\omega \to 0^+$ with hight from 0° to some value trends to $-180°$, indicated by the orange dotted line in fig. S2(d). In this example, this orange dotted vertical line in the Bode phase-frequency diagram does not go across the $-180°$ line. But one may ask if this orange dotted line goes across the $-180°$ line, whether it would change the stability of the system? To make it clear, we change some parameters of $G_3(s)$ to get a new function $G_4(s) = (20/s^2 + 16/s + 11)/[(0.22 \times s + 1)^4 \times (s + 0.4 - 2.5\mathrm{i}) \times (s + 0.4 + 2.5\mathrm{i})]$, and the analyses about $G_4(s)$ are presented in fig. S3(d), (e) and (f). In this new case, the orange dotted line in the Bode phase-frequency diagram goes across the $-180°$ line, as shown in fig. S3(f). Correspondingly, the orange dotted line in the Nyquist diagram also goes across the negative half of the real axis, as shown in fig. S3(d). But because the successive black solid line in the Nyquist diagram goes across the negative half of the real axis, with a direction opposite to that of the orange dotted line, at the left of (-1, 0) point, the crossover of the orange dotted line does not change the times the Nyquist diagram encircles the (-1, 0) point, and hence does not change the stability of the system. Therefore, even if the orange dotted vertical line in the Bode phase-frequency diagram goes across the $-180°$ line, as long as its crossover could be compensated by an opposite crossover of the successive black solid line, this low-frequency-limit crossover does not have any influence on whether the system is stability.

SECTION S2. THE PID CONTROLLER

section S2.1 The transfer function of the PID controller

Ideally the PID controller acts as a PID process, which is composed of the proportion (P), the integration (I) and the differentiation (D) processes. A time-domain expression for the PID process is shown in eq. (S20), in which the error signal $\epsilon(t)$ and output signal $Out_{\mathrm{PID}}(t)$ are the input and output of the PID process respectively, and K_{P}, K_{I} and K_{D} are the coefficients for the proportion, integration and differentiation processes respectively.

$$Out_{\mathrm{PID}}(t) = K_{\mathrm{P}} \times \epsilon(t) + K_{\mathrm{I}} \int_0^t \epsilon(t^{'})\mathrm{d}t^{'} + K_{\mathrm{D}} \times \frac{\mathrm{d}\epsilon(t)}{\mathrm{d}t}. \quad \text{(S20)}$$

The PID process is assumed to start from time 0, so that the integration starts from $t^{'} = 0$. As the PID process

is also a SISO LTI system, it can also be described by a differential equation. By imposing time differentiation on both sides of eq. (S20), we will get

$$\frac{\mathrm{d}Out_{\mathrm{PID}}(t)}{\mathrm{d}t} = K_{\mathrm{P}} \times \frac{\mathrm{d}\epsilon(t)}{\mathrm{d}t} + K_{\mathrm{I}}\epsilon(t) + K_{\mathrm{D}} \times \frac{\mathrm{d}^2\epsilon(t)}{\mathrm{d}t^2}. \quad \text{(S21)}$$

Comparing eq. (S21) with eq. (S1), the general differentiation equation of a SISO LTI system, and eq. (S7), the corresponding transfer function of the SISO LTI system, we can define the transfer function of the PID process as

$$\begin{aligned} FB_{\mathrm{PID}}(s) &= \frac{\mathcal{L}\left[Out_{\mathrm{PID}}(t)\right]}{\mathcal{L}\left[\epsilon(t)\right]} \\ &= \frac{K_{\mathrm{P}} \times s + K_{\mathrm{I}} + K_{\mathrm{D}} \times s^2}{s} \\ &= K_{\mathrm{P}} + \frac{K_{\mathrm{I}}}{s} + K_{\mathrm{D}} \times s. \end{aligned} \quad \text{(S22)}$$

We can also get the transfer function $FB_{\mathrm{PID}}(s)$ by directly applying the Laplace transformation to both sides of eq. (S20). With the help of the Laplace transformations of differentials, see eq. (S4), and the Laplace transformations of integrals, see eq. (S23)

$$\mathcal{L}\left[\int_0^t x(t^{'})\mathrm{d}t^{'}\right] = \frac{\mathcal{L}\left[x(t)\right]}{s}, \quad \text{(S23)}$$

we will find that

$$\mathcal{L}\left[Out_{\mathrm{PID}}(t)\right] = \mathcal{L}\left[\epsilon(t)\right] \times \left(K_{\mathrm{P}} + \frac{K_{\mathrm{I}}}{s} + K_{\mathrm{D}} \times s\right), \quad \text{(S24)}$$

from which we will also get the expression of the transfer function $FB_{\mathrm{PID}}(s)$, same as eq. (S22).

In fact, eq. (S22) is the transfer function of an ideal PID process and is not physically realizable, because it is contradicted to requirement of the physically realizable system, i.e., the number of zeros of the transfer function should not exceed the number of poles. This physically unrealizable property comes from the differentiation. As an example, if the input is the step function $1(t)$, the output of the differentiation will be the Dirac delta function and will be infinite at $t = 0$, which is not realizable in a realistic system. In a practical PID controller, its transfer function can be approximated as [5]

$$FB_{\mathrm{PID}}(s) \approx K_{\mathrm{P}} + \frac{K_{\mathrm{I}}}{s} + \frac{K_{\mathrm{D}} \times s}{1 + s \times T}, \quad \text{(S25)}$$

in which the differentiation is modified by multiplying by a low-pass filter $1/(1 + s \times T)$. As a result, eq. (S25) has equal number of zeros and poles and is physically realizable. Typically, T is very small and this modification only affects the performance in the high frequency range. As long as $|s \times T|$ is much smaller than 1 in the frequency range we are concerned, the effect from this modification can be neglected and we can still use the ideal PID transfer function eq. (S22) to analyze the performance of the magnetic-field stabilization system.

section S2.2 A typical Bode diagram of the PID controller

For better understanding how the PID controller deals with input signals of different frequencies, we can plot the Bode diagrams of its transfer function $FB_{\mathrm{PID}}(s)$, through setting $\sigma = 0$, or $s = \mathrm{i}\omega$, and then plotting the amplitude and phase of $FB_{\mathrm{PID}}(s)$ as a function of ω, known as the Bode amplitude-frequency diagram and the Bode phase-frequency diagram respectively. A typical example is shown in fig. S4, with $K_{\mathrm{P}} = 10^2$, $K_{\mathrm{I}} = 10^{-2}$ s^{-1} and $K_{\mathrm{D}} = 10^{-2}$ s.

In the Bode amplitude-frequency diagram fig. S4(a), the black solid line indicates the Bode amplitude-frequency diagram of the PID process, and the blue dash-dotted line, red dashed line and orange dotted line indicate the contributions from the proportion, integration and differentiation processes respectively. The contribution from the proportion process is a horizontal straight line at hight K_{P}. Given that fig. S4(a) is plotted as logarithmic scales for both the horizontal and vertical axis, the contribution from the integration process is a sloped line with a negative slope, which indicates that its amplitude, or the gain of the input signal, is doubled for each twofold decrease in the signal frequency. The integration-related line intersects with the horizontal line of hight 1 at the angular frequency $\omega = K_{\mathrm{I}}$, which is exactly the integration coefficient, as $|K_{\mathrm{I}}/(\mathrm{i}\omega)| = 1$ at this angular frequency. This property can help us figure out K_{I} from

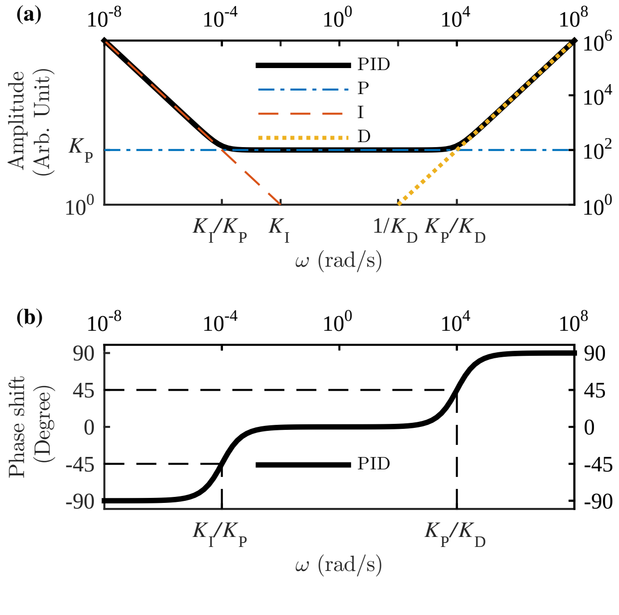

FIG. S4. **A typical Bode diagram of the PID controller.** (a) Bode amplitude-frequency diagram; (b) Bode Phase-frequency diagram. The black solid line shows the Bode diagram of a PID controller, with $K_{\mathrm{P}} = 10^2$, $K_{\mathrm{I}} = 10^{-2}$ s^{-1} and $K_{\mathrm{D}} = 10^{-2}$ s. The contributions from the proportion, integration and differentiation processes on the amplitude are drawn separately in (a).

the Bode diagram of the PID controller. Besides, the proportion-related line and the integration-related line intersect at the angular frequency $\omega = K_{\mathrm{I}}/K_{\mathrm{P}}$, which indicates that the PID controller shown in fig. S4 is dominated by the integration process for the angular frequency smaller than $K_{\mathrm{I}}/K_{\mathrm{P}}$, as the contribution from the integration process is larger than that from the proportion and differentiation processes in this frequency range. Similarly, the contribution from the differentiation process is a sloped line with a positive slope, which indicates that the amplitude is doubled for each twofold increase in the angular frequency. The differentiation-related line intersects with the horizontal line of hight 1 at the angular frequency $\omega = 1/K_{\mathrm{D}}$, which is exactly the inverse of the differentiation coefficient, as $|K_{\mathrm{D}} \times (\mathrm{i}\omega)| = 1$ at this angular frequency. The proportion-related line and the differentiation-related line intersect at angular frequency $\omega = K_{\mathrm{P}}/K_{\mathrm{D}}$, which indicates that the PID controller shown in fig. S4 is dominated by the differentiation process for the angular frequency larger than $K_{\mathrm{P}}/K_{\mathrm{D}}$, as the contribution from the differentiation process is larger than that from the proportion and integration processes in this frequency range.

As for the Bode phase-frequency diagram shown in fig. S4(b), when $K_{\mathrm{I}}/K_{\mathrm{P}} \ll \omega \ll K_{\mathrm{P}}/K_{\mathrm{D}}$, the PID controller is dominated by the proportion process and the phase shift is 0. For the lower frequency range, a negative phase shift is introduced by the integration process, and the phase shift becomes $-45°$ at $\omega = K_{\mathrm{I}}/K_{\mathrm{P}}$, which is the intersection frequency of the proportion and integration related lines in fig. S4(a), and eventually approaches $-90°$ in the low frequency shift. For higher frequency range, the situation is opposite to that in the lower frequency range. A positive phase shift is introduced by the differentiation process in the higher frequency range, and the phase shift becomes $45°$ at $\omega = K_{\mathrm{P}}/K_{\mathrm{D}}$, which is the intersection frequency of the proportion and differentiation related lines in fig. S4(a), and eventually approaches $90°$ in the high frequency shift. Note that although $K_{\mathrm{P}}/K_{\mathrm{D}}$ is much larger than $K_{\mathrm{I}}/K_{\mathrm{P}}$ in the example shown in fig. S4, $K_{\mathrm{P}}/K_{\mathrm{D}}$ can actually be approximately equal to or even less than $K_{\mathrm{I}}/K_{\mathrm{P}}$, depending on the value of the proportion, integration and differentiation coefficients. Besides, the relation that phase shifts at $\omega = K_{\mathrm{I}}/K_{\mathrm{P}}$ and $\omega = K_{\mathrm{P}}/K_{\mathrm{D}}$ are $-45°$ and $45°$ respectively only holds when $K_{\mathrm{I}}/K_{\mathrm{P}} \ll K_{\mathrm{P}}/K_{\mathrm{D}}$. But the phase shifts in the low-frequency limit and high-frequency limit are always $-90°$ and $90°$.

According to fig. S4, it is easy to get the change of the frequency characteristics of the PID controller with the PID coefficients. As an example, when K_{I} increases, the integration-related line in fig. S4(a) moves to the right and as a result the negative slope part of the PID amplitude-frequency characteristic in the low frequency range moves to the right as well. Besides, as $K_{\mathrm{I}}/K_{\mathrm{P}}$, the angular frequency where the phase shift is $-45°$, increases, the negative phase-shift part of the PID phase-frequency characteristic in the low frequency range also moves to the right. For other changes of the PID coefficients, the change trend of the Bode diagram can also be similarly obtained by analyzing the changes of the characteristic frequencies marked on the horizontal axis in fig. S4.

SECTION S3. THE PI^{2}D CONTROLLER

section S3.1 The transfer function of the PI^{2}D controller

Ideally the PI^{2}D controller acts as a PI^{2}D process. As shown in eq. (S26), the PI^{2}D process is formed by adding an additional double integration (I^2) into the PID process shown in eq. (S20), in which K_{I^2} is the coefficient for double integration process.

$$\begin{aligned} Out_{\mathrm{PI^2D}}(t) =& K_{\mathrm{P}} \times \epsilon(t) \\ &+ K_{\mathrm{I}^2} \int_0^t \int_0^{t'} \epsilon(t'')\mathrm{d}t''\mathrm{d}t' \\ &+ K_{\mathrm{I}} \int_0^t \epsilon(t')\mathrm{d}t' + K_{\mathrm{D}} \times \frac{\mathrm{d}\epsilon(t)}{\mathrm{d}t}. \end{aligned} \quad \text{(S26)}$$

According to eq. (S23), the Laplace transformation of the double integral is

$$\begin{aligned} \mathcal{L}\left[\int_0^t \int_0^{t'} x(t'')\mathrm{d}t''\mathrm{d}t'\right] &= \frac{\mathcal{L}\left[\int_0^t x(t')\mathrm{d}t'\right]}{s} \\ &= \frac{\mathcal{L}[x(t)]}{s^2}. \end{aligned} \quad \text{(S27)}$$

As a result, if we apply the Laplace transformation to both sides of eq. (S26), the time-domain express of the PI^{2}D process, according to the Laplace transformations of differentials, see eq. (S4), and the Laplace transformations of integrals, see eq. (S23) and (S27), we will find that

$$\begin{aligned} &\mathcal{L}[Out_{\mathrm{PI^2D}}(t)] \\ =&\mathcal{L}[\epsilon(t)] \times \left(K_{\mathrm{P}} + \frac{K_{\mathrm{I}^2}}{s^2} + \frac{K_{\mathrm{I}}}{s} + K_{\mathrm{D}} \times s\right). \end{aligned} \quad \text{(S28)}$$

So that we can define a transfer function $FB_{\mathrm{PI^2D}}(s)$ as

$$\begin{aligned} FB_{\mathrm{PI^2D}}(s) &= \frac{\mathcal{L}[Out_{\mathrm{PI^2D}}(t)]}{\mathcal{L}[\epsilon(t)]} \\ &= K_{\mathrm{P}} + \frac{K_{\mathrm{I}^2}}{s^2} + \frac{K_{\mathrm{I}}}{s} + K_{\mathrm{D}} \times s. \end{aligned} \quad \text{(S29)}$$

section S3.2 A typical Bode diagram of the PI^{2}D controller

Similar to fig. S4, for better understanding the frequency characteristics of the $FB_{\mathrm{PI^2D}}(s)$, we can also set

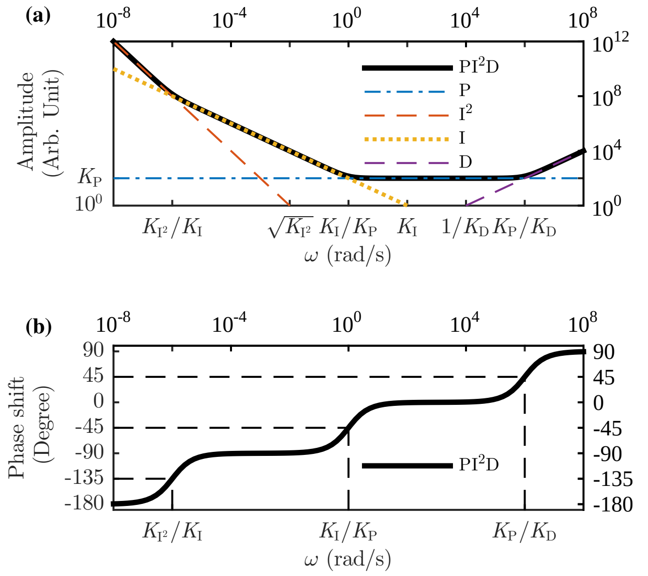

FIG. S5. **A typical Bode diagram of the PI^2D controller.** (a) Bode amplitude-frequency diagram; (b) Bode phase-frequency diagram. The black solid line shows the Bode diagram of a PI^2D controller, with $K_P = 10^2$, $K_{I^2} = 10^{-4}$ s^{-2}, $K_I = 10^2$ s^{-1} and $K_D = 10^{-4}$ s. The contributions from the proportion, double integration, integration and differentiation processes on the amplitude are drawn separately in (a).

$\sigma = 0$, which indicates $s = i\omega$, and then plot the amplitude and phase of $FB_{PI^2D}(s)$ as a function of ω. A typical example is shown in fig. S5, with $K_P = 10^2$, $K_{I^2} = 10^{-4}$ s^{-2}, $K_I = 10^2$ s^{-1} and $K_D = 10^{-4}$ s. Except for the changes induced by the additional double integration process in the low frequency range (blow $\sim 100K_{I^2}/K_I$), the frequency characteristic of the PI^2D controller is almost the same as that of the PID controller shown in fig. S4. In fig. S5(a), the black solid line indicates the Bode amplitude-frequency diagram of the PI^2D controller, and the blue dash-dotted line, red dashed line, orange dotted line and purple dashed line indicate the contributions from the proportion, double integration, integration and differentiation processes respectively. Given that fig. S5(a) is plotted as logarithmic scales for both the horizontal and vertical axis, the contribution from the double integration process is a sloped line with a negative slope, which indicates that the amplitude increases fourfold for each twofold decrease in the angular frequency. The double-integration-related line intersects with the horizontal line of hight 1 at the angular frequency $\omega = \sqrt{K_{I^2}}$, as $|K_{I^2}/(i\omega)^2| = 1$ at this angular frequency. Besides, the I^2-related line and the integration-related line intersect at the angular frequency $\omega = K_{I^2}/K_I$, which indicates that the PI^2D controller shown in fig. S5 is dominated by the double integration process for the angular frequency smaller than K_{I^2}/K_I, as the contribution from the double integration process is larger than that from the integration, proportion and differentiation processes in this frequency range. As for the Bode phase-frequency diagram shown in fig. S5(b), an additional negative phase shift is introduced by the double integration process in the low frequency range, and the phase shift becomes $-135°$ at $\omega = K_{I^2}/K_I$, which is the intersection frequency of the double integration and integration related lines in fig. S5(a), and eventually approaches $-180°$ in the low-frequency limit. Note that although in the example shown in fig. S5 we have $K_{I^2}/K_I \ll K_I/K_P \ll K_P/K_D$, the actually relationship between them is dependent on the value of the proportion, double integration, integration and differentiation coefficients. Besides, the relation that phase shift at $\omega = K_{I^2}/K_I$ is $-135°$ only holds when $K_{I^2}/K_I \ll K_I/K_P$ and $K_{I^2}/K_I \ll K_P/K_D$. But the phase shift in the low-frequency limit is always $-180°$.

According to fig. S5, it is also easy to get the change of the frequency characteristics with the PI^2D coefficients by analyzing the changes of the characteristic frequencies marked on the horizontal axis. As an example, when K_{I^2} increases, the I^2-related line in fig. S5(a) moves to the right and as a result the negative slope part below $\omega = K_{I^2}/K_I$ of the PI^2D amplitude-frequency characteristic moves to the right as well. Besides, as K_{I^2}/K_I, the angular frequency where the phase shift is $-135°$, increases, the additional negative phase-shift part introduced by the double integration process of the PI^2D phase-frequency characteristic also moves to the right. For other changes of the PI^2D coefficients, the change trend of the frequency characteristics can be obtained with similar analysis.

SECTION S4. THE TRANSFER FUNCTION OF THE CLOSED-LOOP AMPLITUDE-MODULATED NONLINEAR MAGNETO-OPTICAL ROTATION MAGNETOMETER

Amplitude-modulated nonlinear magneto-optical rotation (AM NMOR) magnetometer is a highly sensitive OPM that can operate in a finite magnetic field. The basic idea of such OPM is synchronous optical pumping, in which a circularly polarized pump beam is modulated on and off at the Larmor frequency, a frequency proportional to the strength of the magnetic field up to a scaling factor (gyromagnetic ratio), to polarize the atomic spins and synchronize their Larmor precession around the magnetic field. The precessing atomic spins is monitored with a linearly polarized probe beam and the result is demodulated with a lock-in amplifier (LIA). When the modulation frequency is at the nearby of the Larmor frequency, the LIA output is proportional to the modulation detuning, or the difference between modulation frequency and the Larmor frequency, which helps to determine the Larmor frequency and in turn helps to figure out the strength of the magnetic field.

The idea of the closed-loop operation is making the modulation frequency tracking the larmor frequency, so

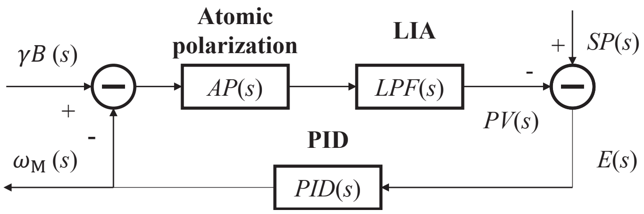

FIG. S6. **Block diagram of the closed-loop operation of the AM NMOR magnetometer.** γ: the gyromagnetic ratio of Cs atoms, $B(s)$: the magnetic field in the Laplace domain, $AP(s)$: the transfer function of the atomic polarization, $LPF(s)$: the transfer function of the LIA, which acts as a low-pass filter, $PV(s)$: the output of the LIA, $SP(s)$: the setpoint in the PID controller, which is 0 throughout the experiment, $E(s)$: the error signal in the PID controller, $PID(s)$: the transfer function of the PID controller, $\omega_M(s)$: the modulation frequency of the pump beam.

that atoms is always in resonance. The closed-loop operation of the AM NMOR magnetometer can be described with the transfer function model shown in fig. S6, in which signals are analyzed in the Laplace domain. As shown in fig. S6, γ is the gyromagnetic ratio of Cs atoms, $B(s)$ is the magnetic field and $\omega_M(s)$ is the modulation frequency of the pump beam. In the open loop operation, the modulation frequency $\omega_M(s)$ is fixed, and the transfer function of the magnetometer is $AP(s) \times LPF(s)$, in which $AP(s)$ and $LPF(s)$ are the transfer functions of the atomic spins and the LIA respectively. In the closed-loop operation, the open loop output is compared with the setpoint, which is 0 throughout the experiment, and their difference serves as the error signal $E(s)$ in PID controller.

$$E(s) = -\big(\gamma B(s) - \omega_M(s)\big) \times AP(s) \times LPF(s). \quad \text{(S30)}$$

The output of the PID controller feedback controls the modulation frequency $\omega_M(s)$, so the modulation frequency of pump beam is

$$\begin{aligned}\omega_M(s) &= E(s) \times PID(s) \\ &= \big(\omega_M(s) - \gamma B(s)\big) \times AP(s) \times LPF(s) \times PID(s).\end{aligned}$$

As the feedback controller controls the modulation frequency in a manner to suppress the error signal to 0, the modulation frequency actively tracks the Larmor frequency. As a result, the modulation frequency can serve as the readout of the closed-loop operation. By rearrange the equation we get the frequency response of the closed-loop operation

$$\begin{aligned}FR(s) &= \frac{\omega_M(s)}{\gamma B(s)} \\ &= 1 + \frac{1}{AP(s) \times LPF(s) \times PID(s) - 1}.\end{aligned} \quad \text{(S31)}$$

To get more specific expression of the frequency response, we need the expression of $AP(s)$, $LPF(s)$ and $PID(s)$ respectively. When the magnetometer is near resonance, in another word, when the modulation detuning $|\gamma B - \omega_M|$ is much smaller than the atomic relaxation rate $1/\tau$, in which τ is the transverse relaxation time, the transfer function of the atomic spins can be approximated as a first-order Butterworth low-pass filter [6]:

$$AP(s) \approx \frac{1}{1 + \tau s}. \quad \text{(S32)}$$

The transfer function of LIA is determined by the low-pass filter in LIA, which is a q-order Butterworth low-pass filter in our LIA. So the transfer function of the LIA is

$$LPF(s) = \frac{1}{(1 + \Gamma s)^q}, \quad \text{(S33)}$$

where Γ is the time constant and q is the order of the filter. According to eq. (S22), the transfer function of the PID controller is

$$PID(s) = K_p + \frac{K_i}{s} + K_d \times s, \quad \text{(S34)}$$

in which K_p, K_i and K_d are proportion, integration and differentiation coefficients respectively. When we substitute eq. (S32), (S33) and (S34) into (S31), given that $K_i \neq 0$ and $K_d \neq 0$, we will find that in the low-frequency limit,

$$FR(s)|_{s\to 0} = 1, \quad \text{(S35)}$$

and that in the high-frequency limit,

$$FR(s)|_{s\to\infty} \propto \frac{1}{s^q}. \quad \text{(S36)}$$

In our experiment, $q = 4$, so that

$$FR(s)|_{s\to\infty} \propto \frac{1}{s^4}. \quad \text{(S37)}$$

SECTION S5. PARAMETERS OPTIMIZATION OF THE MAGNETIC-FIELD STABILIZATION SYSTEM

To efficiently optimize the performance of the magnetic-field stabilization system, it is better to understand how the variations of the system parameters influence the stability and the noise rejection ratio of this system. Here we take a stabilization system with the PID module as an example. We adopt the method we used in Fig. 2 in the main text to analyze how the variations of the PID coefficients and magnetometer's bandwidth influence the performance of this system, and we give a brief parameters-adjustment guide. The analyses of the magnetic-field stabilization system with PI^{2}D modules or some other feedback control modules can be done in a similar way.

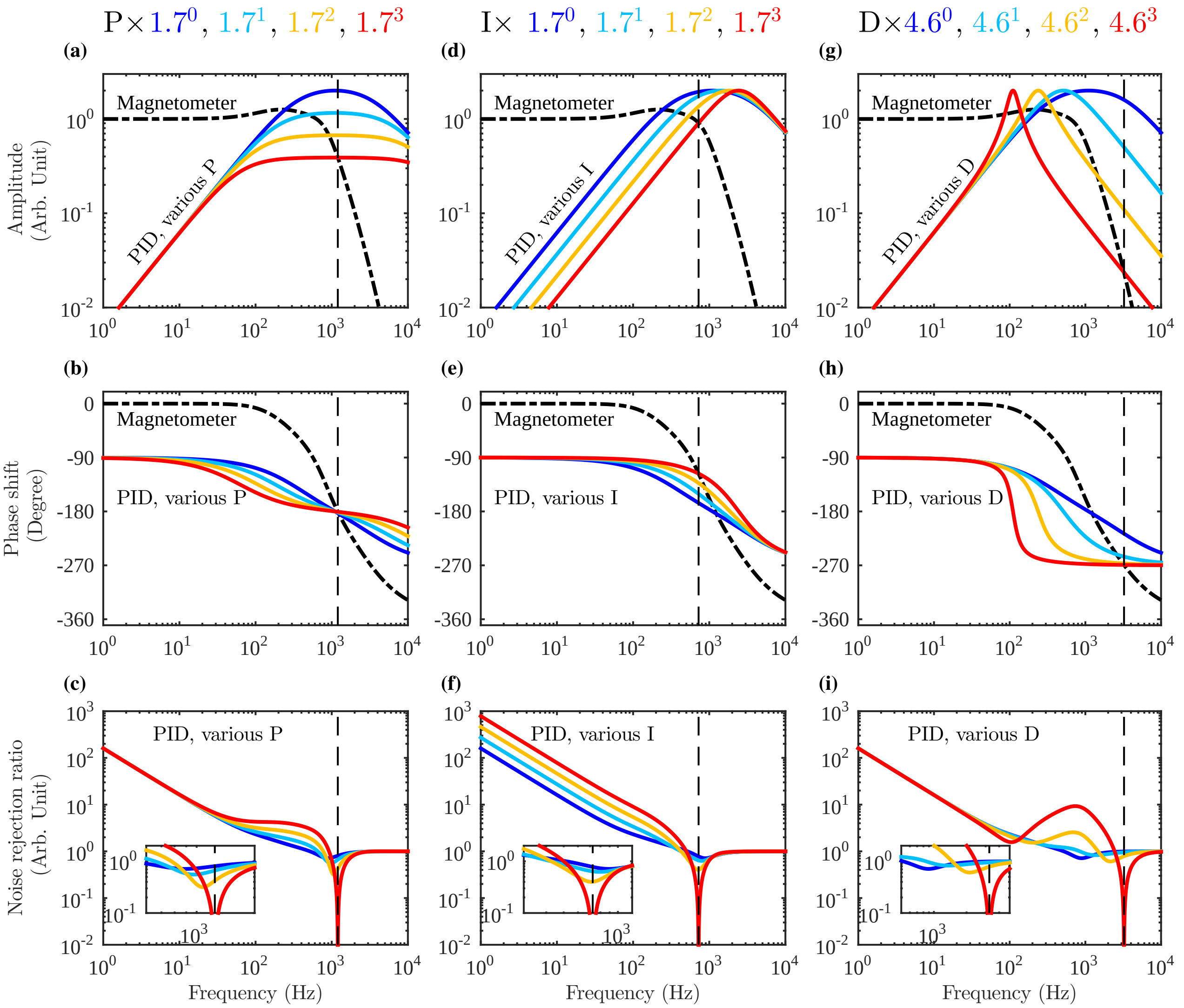

FIG. S7. **The influence of the PID coefficients on the the magnetic-field stabilization system.** The sub-figures in the left, the central and the right columns indicate the results for variations on the proportion, integration, and differentiation coefficients respectively. The sub-figures in the the top, the medium and the bottom rows indicate the Bode amplitude-frequency diagrams, the Bode phase-frequency diagrams and the noise rejection ratios respectively. The dashed-dotted black lines in the sub-figures in the the top and the medium rows are the calculated amplitudes and phases of the frequency responses of the magnetometer respectively. The solid lines in the sub-figures in the the top and the medium rows are the calculated amplitudes and phases of the frequency responses of $-1/[FB(s) \times MG(s)]$ respectively. The different colors of the solid lines indicate different PID coefficients, in which from blue to red the coefficients increase. The solid lines in the sub-figures in the the bottom row are the calculated noise rejection ratios corresponding to different PID coefficients. The insets in the sub-figures in the the bottom row present details about the noise rejection ratios near the frequency where the red lines approach infinitesimal.

section S5.1 The influence of feedback coefficients on the magnetic-field stabilization system

Here we analyze how the stabilization system is influenced when the proportion, integration and differentiation coefficients are changed individually, and the results are shown in the left, the central and the right columns in fig. S7 respectively. The top and medium rows show the Bode amplitude-frequency diagrams and the Bode phase-frequency diagrams respectively, in which the calculated $FR(s)$ and $-1/[FB(s)\times MG(s)]$ are presented. The bottom row shows the noise rejection ratios corresponding to different PID coefficients.

Generally speaking, the increases of the PID coefficients initially lead to increase of the noise rejection ratios, like from the deep blue lines to the light blue lines, and the increases of the proportion, integration and the differentiation coefficients are mainly responsible for the

increases of noise rejection ratios in the low, the medium and the high frequency ranges respectively.

But when the PID coefficients are further increased, the stabilities of the systems start to be sacrificed, and finally the systems become unstable, as shown in the red lines. According to the Bode-diagram based stability criterion, a system is stable when $|FR(s)\times FB(s)\times MG(s)|$ is smaller than 1 at the frequency where the phase of $FR(s)\times FB(s)\times MG(s)$ is $-180°$. As we discussed in the main text, for a system shown in fig. S7, the stability criterion means that this system is stable when the frequency where the amplitudes of $FR(s)$ and $-1/[FB(s)\times MG(s)]$ intersect is smaller than the frequency where the phases of $FR(s)$ and $-1/[FB(s)\times MG(s)]$ intersect. When we further increase the PID coefficients, the distances between these two kinds of intersections get smaller, and the noise rejection ratios are decreased in some frequency ranges. When it comes to the red lines, the amplitude-intersection frequencies and the phase-intersection frequencies coincide with each other, which are marked by the vertical dashed lines, and the corresponding noise rejection ratios in the bottom rows approach infinitesimal at the intersection frequencies. This means that the systems represented by the red lines are at the stability limit and there are non-decay oscillations in their outputs at intersection frequencies.

If we look closer at the changes of each coefficient, we will find that they have different effects on the stability of the system. The increase of the proportion coefficient mainly increases the amplitude-intersection frequency, and has little influence on the phase-intersection frequency. The increase of the integration coefficient increases the amplitude-intersection frequency and decreases the phase-intersection frequency at the same time. When the differentiation coefficient is increased, both the amplitude and phase-intersection frequencies increase, but the increase of the amplitude-intersection frequency is speeded up, while the increase of the phase-intersection frequency is slowed down. As a result, the distance between these two intersection frequencies is getting larger at the beginning and then getting smaller.

To conclude, as we are interested in the stabilization performance in the low frequency range, we need a large integration coefficient. Besides, modest increase of the differentiation coefficient is beneficial for the stability of the system. But non of the PID coefficients can be increased without limit, otherwise the system will become unstable. If a more accurate parameters adjustment is in demand, one can do some calculations based on this model.

section S5.2 The influence of magnetometer's bandwidth on the magnetic-field stabilization system

From last section we see that the enhancement of the noise rejection ratio is limited by the stability requirement of the system. Actually, if the magnetometer has a broader bandwidth, we can further improve the noise rejection ratio without sacrificing the stability of the system. In this section we talk about how to optimize the magnetic-field stabilization system if we have a magnetometer of broader bandwidth.

Given that the initial transfer functions of the magnetometer, the feedback controller and the magnetic-field generator are $FR(s)$, $FB_{\rm PID}(s)$ and $MG(s)$ respectively. Then we extend the magnetometer's bandwidth by N times and get a new transfer function $FR'(s)$. If the shape of $FR'(s)$ is similar to that of $FR(s)$, an approximate expression of the new transfer function $FR'(s)$ is the frequency scaling of the original transfer function $FR(s)$:

$$FR'(s)\approx FR(s/N). \tag{S38}$$

As the frequency response of the magnetometer is changed, we should also change the parameters of our feedback controller to optimize the system performance. In our system, the bandwidth of the magnetic-field generator is much wider than the frequency range we are concerned, so $MG(s)$ is approximated to 1 during the following analyses. We can optimize the feedback controller in a manner that $K'_{\rm P}=K_{\rm P}$, $K'_{\rm I}=N\times K_{\rm I}$ and $K'_{\rm D}=K_{\rm D}/N$, in which $K_{\rm P,I,D}$ are the original PID coefficients and $K'_{\rm P,I,D}$ are the new coefficients. As a result, the new transfer function of the feedback controller is

$$\begin{aligned}FB'_{\rm PID}(s)&=K'_{\rm P}+\frac{K'_{\rm I}}{s}+K'_{\rm D}\times s\\&=K_{\rm P}+N\times\frac{K_{\rm I}}{s}+\frac{K_{\rm D}\times s}{N}\\&=K_{\rm P}+\frac{K_{\rm I}}{s/N}+K_{\rm D}\times\frac{s}{N}\\&=FB_{\rm PID}(s/N),\end{aligned}\tag{S39}$$

which means that the new transfer function of the feedback controller is also the frequency scaling of its original transfer function. According to definition of the transfer function of the whole magnetic-field stabilization system, see eq. (S18), the new transfer function of the stabilization system is

$$\begin{aligned}H'(s)&=\frac{1}{1+FR'(s)\times FB'_{\rm PID}(s)\times MG(s)}\\&\approx\frac{1}{1+FR'(s)\times FB'_{\rm PID}(s)}\\&\approx\frac{1}{1+FR(s/N)\times FB_{\rm PID}(s/N)}\\&\approx H(s/N).\end{aligned}\tag{S40}$$

Consequently, the new transfer function of the stabilization system is also the frequency scaling of its original transfer function, which means that except for a N times extension in the bandwidth, the new stabilization system inherits almost all the features of the previous system, and of course the new system is also stable as long as the previous one is stable.

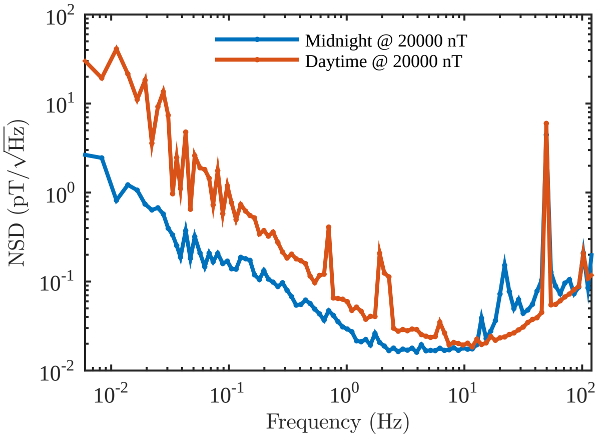

FIG. S8. **Noise spectral density at different time.** Blue (Red) line represents the NSD of the gradiometer at midnight (daytime) at 20000 nT.

Now we are going to consider the scaling of the noise rejection ratio induced by this frequency extension. Assume that we are considering the noise rejection ratio at a fixed frequency, for example, at 10 Hz in fig. S7(c). In the nearby of that frequency, the noise rejection ratio approximates to a straight line with a negative slope, and the noise rejection ratio is N times increased for every N times decrease of frequency. As a result, relationship between the noise rejection ratios of the new system, or $NRR'(s)$, and that of the previous system, or $NRR(s)$ is

$$\begin{aligned} NRR'(s) &= \frac{1}{|H'(s)|} \approx \frac{1}{|H(s/N)|} \\ &= NRR(s/N) \approx N \times NRR(s). \end{aligned} \tag{S41}$$

Consequently, the noise rejection ratio is enhanced by the same amount of the magnetometer's bandwidth extension ratio.

SECTION S6. THE NOISE FLOOR OF THE MAGNETOMETER

The noise spectral densities (NSD) of the gradiometer at midnight and daytime at 20000 nT are compared in fig. S8. It is clear that the NSD at midnight is smaller than that of the daytime for frequency smaller than 10 Hz, and the slope that NSD increases with the decreased frequency is also smaller at midnight. The reason is that for frequency lower than 10 Hz, the NSD at daytime is limited by the magnetic gradient noise. At midnight, the NSD is doubled for around each twofold decrease in the frequency for frequency lower than 2 Hz, which indicates that the NSD is dominated by the $1/f$ noise of magnetometers themselves.

[1] L. Keviczky, R. Bars, J. Hetthéssy, and C. Bányász, Description of continuous linear systems in the time, operator and frequency domain, in *Control engineering* (Springer Singapore, 2019) pp. 37–126.

[2] L. Keviczky, R. Bars, J. Hetthéssy, and C. Bányász, Stability of linear control systems, in *Control engineering* (Springer Singapore, 2019) pp. 197–239.

[3] A. Bacciotti, *Stability and control of linear systems* (Springer, Cham, 2019).

[4] L. Keviczky, R. Bars, J. Hetthéssy, and C. Bányász, Proofs and derivations (by chapters), in *Control engineering* (Springer Singapore, 2019) pp. 37–126.

[5] L. Keviczky, R. Bars, J. Hetthéssy, and C. Bányász, Design of conventional regulators, in *Control engineering* (Springer Singapore, 2019) pp. 277–324.

[6] R. Zhang, B. Pang, W. Li, Y. Yang, J. Chen, X. Peng, and H. Guo, Frequency response of a close-loop bell-bloom magnetometer, in *2018 IEEE International Frequency Control Symposium (IFCS)* (2018) pp. 1–3.
